# Blocking Junctional Adhesion Molecule C Enhances Dendritic Cell Migration and Boosts the Immune Responses against *Leishmania major*

**DOI:** 10.1371/journal.ppat.1004550

**Published:** 2014-12-04

**Authors:** Romain Ballet, Yalin Emre, Stéphane Jemelin, Mélanie Charmoy, Fabienne Tacchini-Cottier, Beat A. Imhof

**Affiliations:** 1 Department of Pathology and Immunology, Centre Médical Universitaire, University of Geneva, Geneva, Switzerland; 2 Department of Biochemistry, and WHO Immunology Research and Training Center, University of Lausanne, Epalinges, Switzerland; Imperial College London, United Kingdom

## Abstract

The recruitment of dendritic cells to sites of infections and their migration to lymph nodes is fundamental for antigen processing and presentation to T cells. In the present study, we showed that antibody blockade of junctional adhesion molecule C (JAM-C) on endothelial cells removed JAM-C away from junctions and increased vascular permeability after *L. major* infection. This has multiple consequences on the output of the immune response. In resistant C57BL/6 and susceptible BALB/c mice, we found higher numbers of innate immune cells migrating from blood to the site of infection. The subsequent migration of dendritic cells (DCs) from the skin to the draining lymph node was also improved, thereby boosting the induction of the adaptive immune response. In C57BL/6 mice, JAM-C blockade after *L. major* injection led to an enhanced IFN-γ dominated T helper 1 (Th1) response with reduced skin lesions and parasite burden. Conversely, anti JAM-C treatment increased the IL-4-driven T helper 2 (Th2) response in BALB/c mice with disease exacerbation. Overall, our results show that JAM-C blockade can finely-tune the innate cell migration and accelerate the consequent immune response to *L. major* without changing the type of the T helper cell response.

## Author Summary

Leishmaniasis is a parasitic disease transmitted to humans through sand fly bites. Clinical symptoms vary from self-healing cutaneous lesions to death. Cutaneous leishmaniasis is particularly studied in mice inoculated with *Leishmania major*. In this model, some strains (e.g. C57BL/6) are resistant due to a Th1 immune response promoting parasite killing. Conversely, other strains (e.g. BALB/c) are susceptible due to a nonprotective Th2 response. DCs are professional antigen-presenting cells that educate antigen-specific T cells. Improving the migration of DCs from the site of infection to the lymph nodes, where T cells reside, may improve the T cell response. JAM-C is a vascular adhesion molecule implicated in leukocyte migration in different inflammatory models. We found that JAM-C blockade with antibodies increases vascular permeability and consequently improves the migration of DCs to sites of infection and draining lymph nodes. This increased leukocyte migration boosted the induction of the Th1 response in resistant mice, while in susceptible mice the Th2 response was augmented. This led to disease improvement or exacerbation, respectively. Our results illustrate the key role of a vascular adhesion molecule in controlling leukocyte migration and the subsequent immune events in response to pathogen infections.

## Introduction

*Leishmania* is an obligate intracellular parasite responsible for a wide spectrum of clinical manifestations, such as cutaneous, mucocutaneous or visceral leishmaniasis [1]. After inoculation of *Leishmania major* in the skin of humans or rodents, promastigotes are taken up by phagocytic cells [2]. The infection leads to the development of cutaneous lesions, which eventually heal depending on the adaptive immune response of the host [3]. In the C57BL/6 mouse model, resistance to *L. major* infection is associated with the production of IFN-γ by CD4^+^ Th1 lymphocytes [4], [5]. The secretion of IFN-γ by these Th1 cells then activates infected macrophages, and leads to efficient killing of the parasites [2], [6]. Conversely, BALB/c mice mount a non-protecting T helper 2 response (Th2) characterized by production of anti-inflammatory cytokines such as IL-4, IL-10, and IL-13 [3], [7].

Dendritic cells (DCs) are professional antigen-presenting cells that play a key role in the induction of the adaptive immune reaction against *L. major*. At early stages of infection in C57BL/6 mice, resident dermal DCs phagocytose the parasites [8] and promote the switch towards a Th1 response by producing IL-12 [9]. Monocytes, subsequently recruited to the site of infection can also give rise to monocyte-derived DCs (mo-DCs). During the late phase of infection, such mo-DCs are essential mediators of the protective T cell response. They efficiently migrate from the site of infection to the draining lymph node, where they induce a specific immune reaction against the pathogen [10]. The fundamental role of monocytes and mo-DCs has been further highlighted with the use of the CCR2 knock-out in the C57BL/6 background. In these mice, the recruitment of mo-DC to the lymph nodes is severely reduced, diminishing the Th1 cells [11], and resulting in a non-healing phenotype similar to that observed in susceptible mice [12]. Therefore, migration of DCs to the infected skin and lymph node can be considered as fundamental steps towards immunity against *L. major*.

Transendothelial migration of leukocytes from blood to the site of inflammation is a complex process controlled by adhesion molecules, such as PECAM-1, ICAM-2, ICAM-1, CD99, ESAM, or junctional adhesion molecules (JAMs) [13]. The JAM family is composed of 6 molecules comprising the classical JAM-A, JAM-B, and JAM-C, mainly localized in the tight junctions of endothelial cells [14]. In humans, JAM-C is also found on subpopulations of T and B lymphocytes, and platelets [15], [16], while murine JAM-C is restricted to endothelial and stromal cells [17]–[19]. In the steady state, JAM-C mainly interacts with JAM-B [20] at cell-cell contacts. Moreover, JAM-C and JAM-B can also bind the integrins α_M_β_2_ (Mac-1) and α_4_β_1_ (VLA-4), respectively [16], [21].

We previously described a monoclonal antibody raised against mouse JAM-C, namely H33 [22]. H33 blocks JAM-C/JAM-B interaction and redistributes JAM-C away from tight junctions [20]. Interestingly, redistribution of JAM-C on the apical side of endothelial cells makes it available for interactions with its counter-receptor α_M_β_2_, an integrin found on neutrophils and monocytes, therefore increasing their adhesion on endothelial cells [20]. More recently, it was shown that H33 increases reverse and repeated transmigration of monocytes and neutrophils, in mouse models of peritonitis, and ischemia reperfusion injury, respectively [23], [24]. However, the role of endothelial JAM-C in leukocyte migration in the context of infectious disease was not addressed yet.

In this report, we studied the involvement of JAM-C in leukocyte trafficking and the subsequent immune response against *L. major* infection. We first observed that JAM-C expression by vascular endothelial cells is down regulated after infection with *L. major* at a time window when inflamed endothelium modulates and redistributes its network of junctional proteins for leukocyte transmigration [25]. To dissect the mechanism of JAM-C action in this infectious disease model, we used the antibody H33 to mimic the modulation of JAM-C observed after infection. Strikingly, blocking JAM-C after *L. major* infection *in vivo* increased vascular permeability and promoted leukocyte recruitment to the inflamed tissue, and DC migration to the draining lymph node. More importantly, sustained JAM-C blockade boosted the immune response in both resistant C57Bl/6 and susceptible BALB/c mice. On one hand, H33 treatment improved the IFN-γ-dominated Th1 response in resistant animals, together with decreased lesion size and parasite burden. On the other hand, JAM-C blockade boosted the IL-4-dominated Th2 response in susceptible mice, resulting in disease exacerbation. Collectively, our results show that JAM-C blockade potentiates the immune responses to pathogen infections by improving leukocyte migration.

## Results

### The antibody H33 mimics JAM-C downregulation after *L. major* inoculation, and locally increases vascular permeability after infection

**Figure 1:**
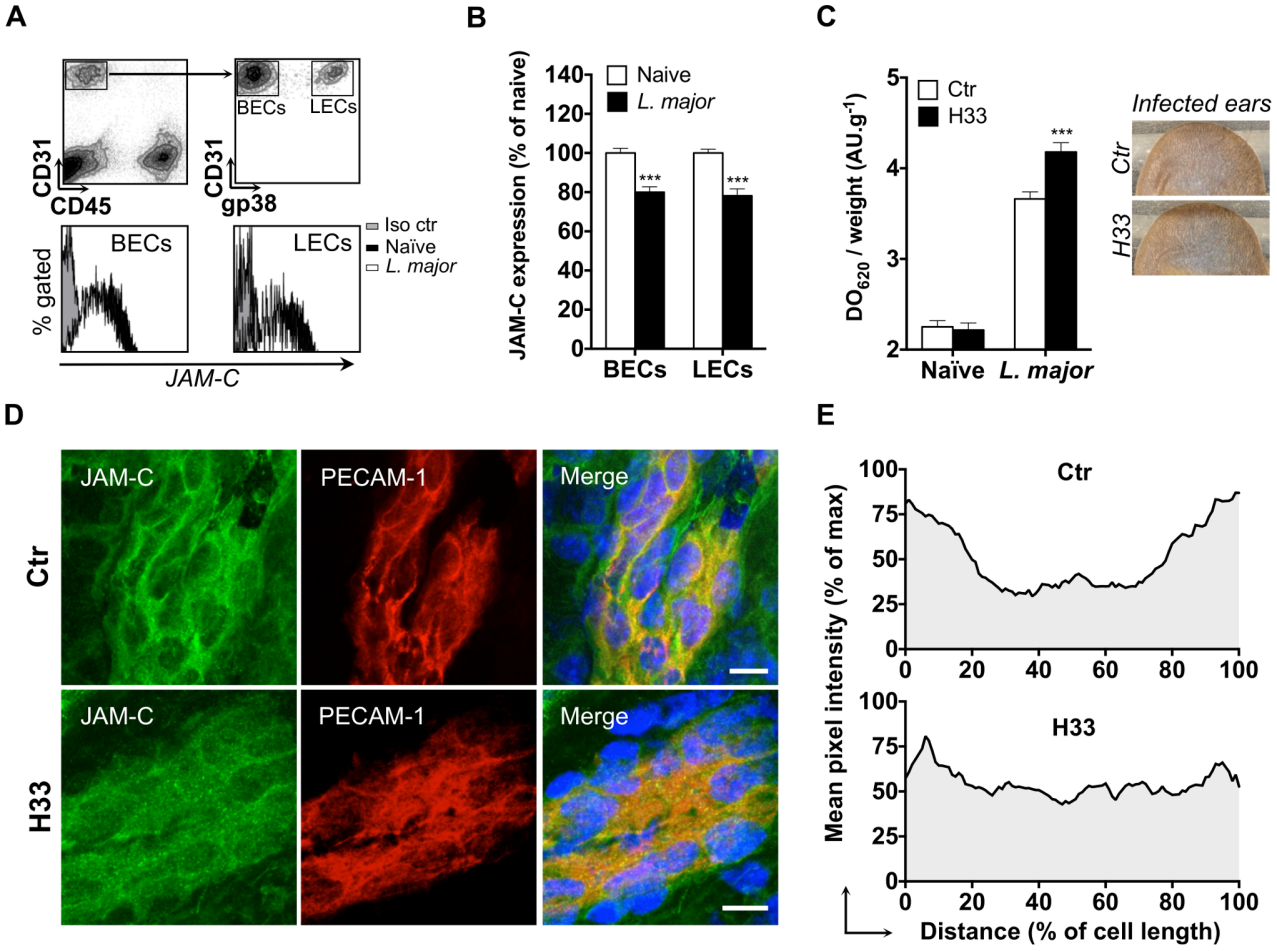
The antibody H33 mimics JAM-C downregulation after L. major inoculation, and locally increases vascular permeability after infection. (A) JAM-C levels in endothelial cells populations of mouse ear. Ears were enzymatically digested and stained for FACS analysis. CD45^−^ CD31^+^ gp38^−^ cells represent blood endothelial cells (BECs), whereas CD45^−^ CD31^+^ gp38^+^ cells are lymphatic endothelial cells (LECs). For each population, a representative histogram overlay is shown with JAM-C in endothelial cells from naïve ears (black filled), JAM-C in endothelials cells from *L. major* infected ears (blank filled), and the isotype control staining (grey filled). (B) The median fluorescence intensity (MFI) of JAM-C in naïve mouse ears (white bars) versus *L. major* infected mouse ears (black bars) was measured in BECs and LECs. The Y-axis scale represents MFI normalized to the mean MFI of naïve ears. Data represent the mean ± SEM of ten individual mice pooled from two separate experiments, and were analyzed by the unpaired Student's t test with ***: p<0.001. (C) Mice were treated with H33 or control antibody 2 hours before Evans blue was injected i.v. and *L. major* inoculated i.d. in the ear dermis. Skin permeability was assessed by the absorbance of Evans blue extracted from the sample normalized to the weight of ear. Results are shown for naïve versus *L. major* infected animals treated with H33 (black bars) or control antibody (blank bars). Representative ear pictures are shown. Data represent the mean ± SEM of seventeen mice per group pooled from two separate experiments, and were analyzed by the unpaired Student's t test with ***: p<0.001. (D) Ear sections from control antibody-treated (top panel) or H33-treated mice (bottom panel) were stained for JAM-C (green) and CD31 (red). Nucleus was stained with DAPI (blue). Scale bars, 10 µm. Control staining for JAM-C is shown in Figure S3. (E) The pixel intensity across 10 representative cells of similar size taken from three mice per group was measured and expressed as a percentage of the maximal pixel intensity. Data represent the average profile plot for the 10 cells per group analyzed.

Blood endothelial cells (BECs) and lymphatic endothelial cells (LECs) from the skin of mouse ears were analyzed by flow cytometry. In the steady state, BECs (CD45^−^ CD31^+^ gp38^−^) and LECs (CD45^−^ CD31^+^ gp38^+^) were JAM-C positive as previously described for other organs [14] (Fig. 1A). Conversely, leukocytes recruited to the infected ear following *L. major* inoculation were all JAM-C negative (S1 Figure). We observed a statistically significant decrease of JAM-C expression in BECs and LECs 24 hours after *L. major* infection (Fig. 1A and B). This was not the consequence of tissue injury caused by the needle, as saline injection did not downregulate JAM-C (S2 Figure). Interestingly, previous studies observed a peak of leukocytes migrating to the site of infection at the same time period [26], [27]. Therefore, we postulated that JAM-C downregulation after infection could enhance vascular permeability and therefore promote inflammation and cell migration.

To study the effect of H33 on vascular permeability, we used a modified Miles assay in which mice were injected i.v. with Evan's blue [28]. Evan's blue is a small molecule that binds strongly to albumin. Consequently, this assay indirectly assesses the exudation of plasma into the tissue accounting for vascular permeability. Mice were treated with H33 or the isotype control antibody before injection of Evan's blue and *L. major* inoculation. Strikingly, treatment with H33 significantly increased the amount of Evan's blue that leaked into the inflamed tissue as compared to control. However, we did not observe any change in vascular permeability under steady state conditions (Fig. 1C).

To understand the mechanism leading to the increased vascular permeability, we investigated by immunofluorescence in our system whether H33 redistributes JAM-C out of ear endothelial cell junctions as previously proposed for other organs [20]. In control mice, JAM-C was strongly expressed at the cell border of CD31 positive endothelial cells (Fig. 1D, top panel), resulting in a U-shaped pattern of distribution of the molecule (Fig. 1E, top panel). In H33-treated animals however, JAM-C was removed from endothelial cell junctions (Fig. 1D, bottom panel), as confirmed by the smoothed pattern of distribution of JAM-C (Fig. 1E, bottom panel). Control staining for JAM-C is provided in S3 Figure.

Altogether, we concluded that the blockade of JAM-C with H33 redistributes JAM-C out of junctions, and increases vascular permeability after *L. major* infection.

### Blocking JAM-C increases the number of circulating cells recruited in response to *L. major* infection

**Figure 2:**
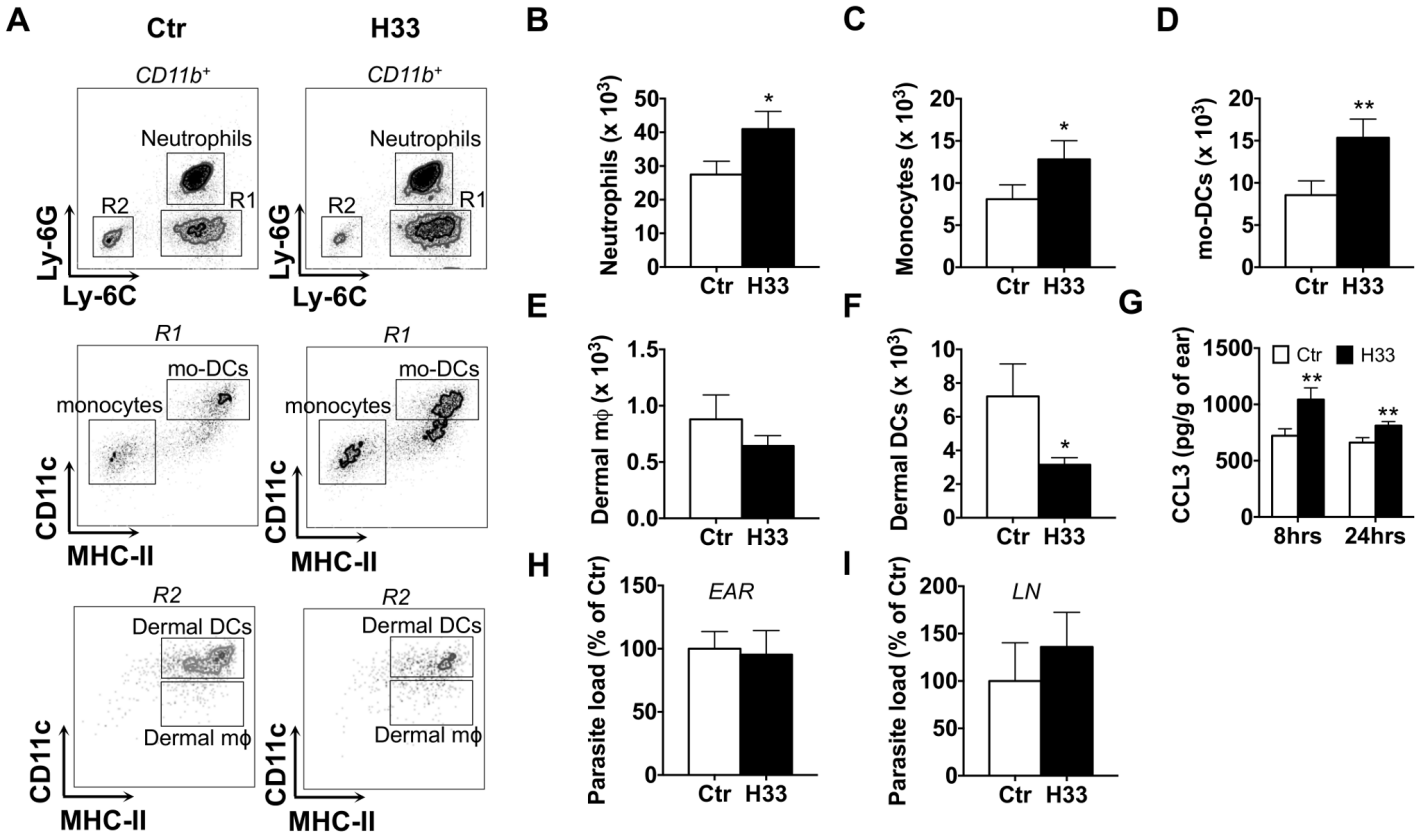
Blocking JAM-C increases the number of leukocytes recruited to the site of *L. major* infection. (A) Representative dot plots of neutrophils (CD11b^+^ Ly6C^+^ Ly6G^+^); monocytes (CD11b^+^ Ly6C^+^ Ly6G^−^ CD11c^−^ IA^−^); mo-DCs (CD11b^+^ Ly6C^+^ Ly6G^−^ CD11c^+^ IA^+^); dermal mφ (CD11b^+^ Ly6C^−^ Ly6G^−^ CD11c^low^ IA^+^); dermal DCs (CD11b^+^ Ly6C^−^ Ly6G^−^ CD11c^high^ IA^+^) in control versus H33-treated animals. (B–F) The number of neutrophils (B), monocytes (C), mo-DCs (D), dermal mφ (E) and dermal DCs (F) was measured in the H33-treated (H33, black bar) versus isotype control-treated mice (Ctr, white bars) 24 hours p.i. Data represent the mean ± SEM of twenty mice per group pooled from 3 separate experiments, and were analyzed by the unpaired Student's t test with *: p<0.05 and **: p<0.01. (G) CCL3 protein levels normalized to the weight of ears were measured in H33-treated (H33, black bar) versus isotype control-treated mice (Ctr, white bars) 8 and 24 hours p.i. Data represent the mean ± SEM of ten mice per group pooled from 2 separate experiments, and were analyzed by the unpaired Student's t test with **: p<0.01. (H–I) The parasite burden in infected ears (H) and draining lymph nodes (LN) (I) were measured 48 hours p.i. by limiting dilution assay (LDA). Data are expressed as a percentage of the mean of the control group ± SEM of ten mice per group pooled from 2 separate experiments, and were analyzed by the unpaired Student's t test. For panel H and I, raw data of one representative experiment are provided in S6 Figure.

To study whether the effect of H33 on vascular permeability potentiates leukocyte recruitment after *L. major* infection, we used wild type C57BL/6 mice treated with H33, and analyzed by FACS the number of emigrating leukocytes 24 hours after infection (Fig. 2A). We observed a significant increase in the numbers of neutrophils, inflammatory monocytes, and mo-DCs in H33-treated animals as compared to control animals (Fig. 2B–D). Meanwhile, the number of non-migrating dermal macrophages (dermal mφ) was not modified (Fig. 2E). Finally, the number of emigrating dermal DCs, a cell type that efficiently migrates to the draining lymph node once activated, was decreased in H33-treated animals (Fig. 2F). In line with the absence of vascular permeability observed in the steady state (Fig. 1F), JAM-C blockade did not increase leukocyte emigration in naïve mouse ears (S4 Figure). Moreover, we found no difference in the number of leukocytes in the bone marrow and in the blood (S5 Figure). This suggests that H33 does neither increase haematopoiesis nor leukocyte emigration from the bone marrow to the blood in normal homeostasis.

We also measured higher levels of the monocytes and mo-DCs attracting chemokine CCL3 in H33 treated animals early after infection (Fig. 2G). This is in line with the increased number of neutrophils, a cell type known to produce CCL3 to attract mo-DCs in response to *L. major* [26]. Interestingly, the higher numbers of innate immune cells recruited with H33 did not impact on the parasite load early after infection (Fig. 2H and S6 Figure). Moreover, the dissemination of the parasites to the draining lymph node was unchanged (Fig. 2I and S6 Figure).

Overall, our data showed that JAM-C blockade with H33 increases leukocyte recruitment to the site of infection, and strongly suggest that H33 may influence DC migration to the draining lymph node.

### Blocking JAM-C increases the number of DCs migrating to the draining lymph node

**Figure 3:**
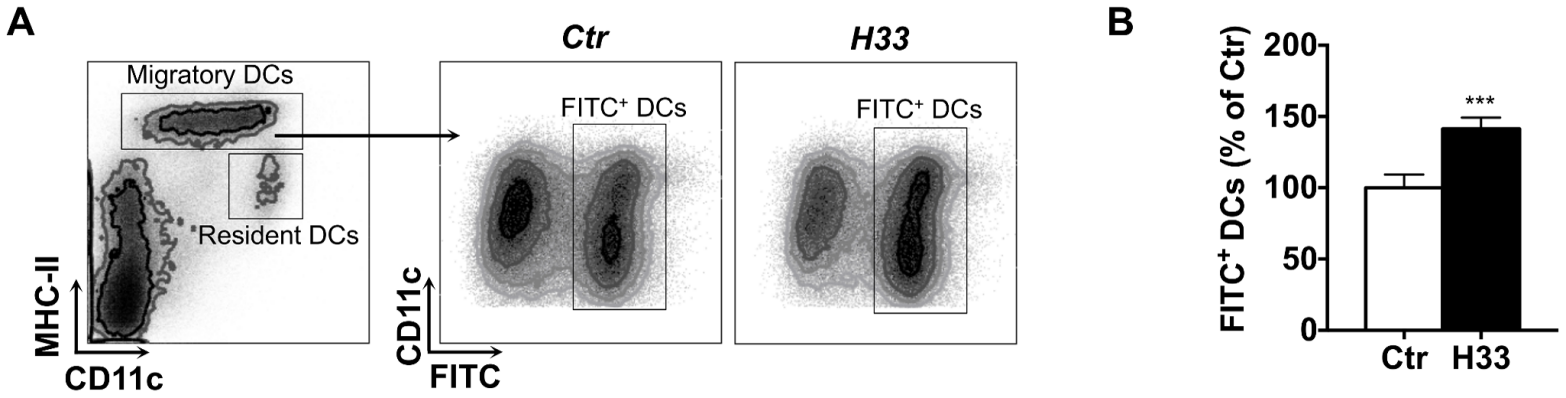
Blocking JAM-C increases the number of DCs migrating to the draining lymph node. (A) The ear draining lymph node cells were harvested and stained for FACS analysis 18 hours after FITC-painting. Representative FACS dot plots are shown. (B) The number of IA^high^ CD11c^+^ FITC^+^ migratory DCs was counted. Data are expressed as a percentage of the mean of the control group ± SEM of eighteen mice per group pooled from 3 separate experiments, and were analyzed by the unpaired Student's t test with ***: p<0.001. Raw data from one representative experiment are provided in S7 Figure.

To investigate the effect of H33 on DC migration to the draining lymph node, we used the FITC painting assay. In this model, migration of dermal and epidermal DCs to lymph nodes is induced and peaks 18 hours after painting [29]. Based on MHC class II (IA) and CD11c, two populations of DCs can be distinguished by FACS in the lymph node: MHC-II^high^ CD11c^+^ migratory DCs, and MHC-II^+^ CD11c^high^ lymphoid resident DCs (Fig. 3A). Strikingly, we found higher numbers of FITC^+^ IA^high^ CD11c^+^ migratory DCs in lymph nodes of H33-treated mice as compared to control animals (Fig. 3A and B, and S7 Figure). Therefore, H33 treatment not only increases leukocyte recruitment to the site of infection, but also increases the migration of DCs to the draining lymph node.

### Blocking JAM-C improves the Th1 cell response and favours healing in C57BL/6 mice

**Figure 4:**
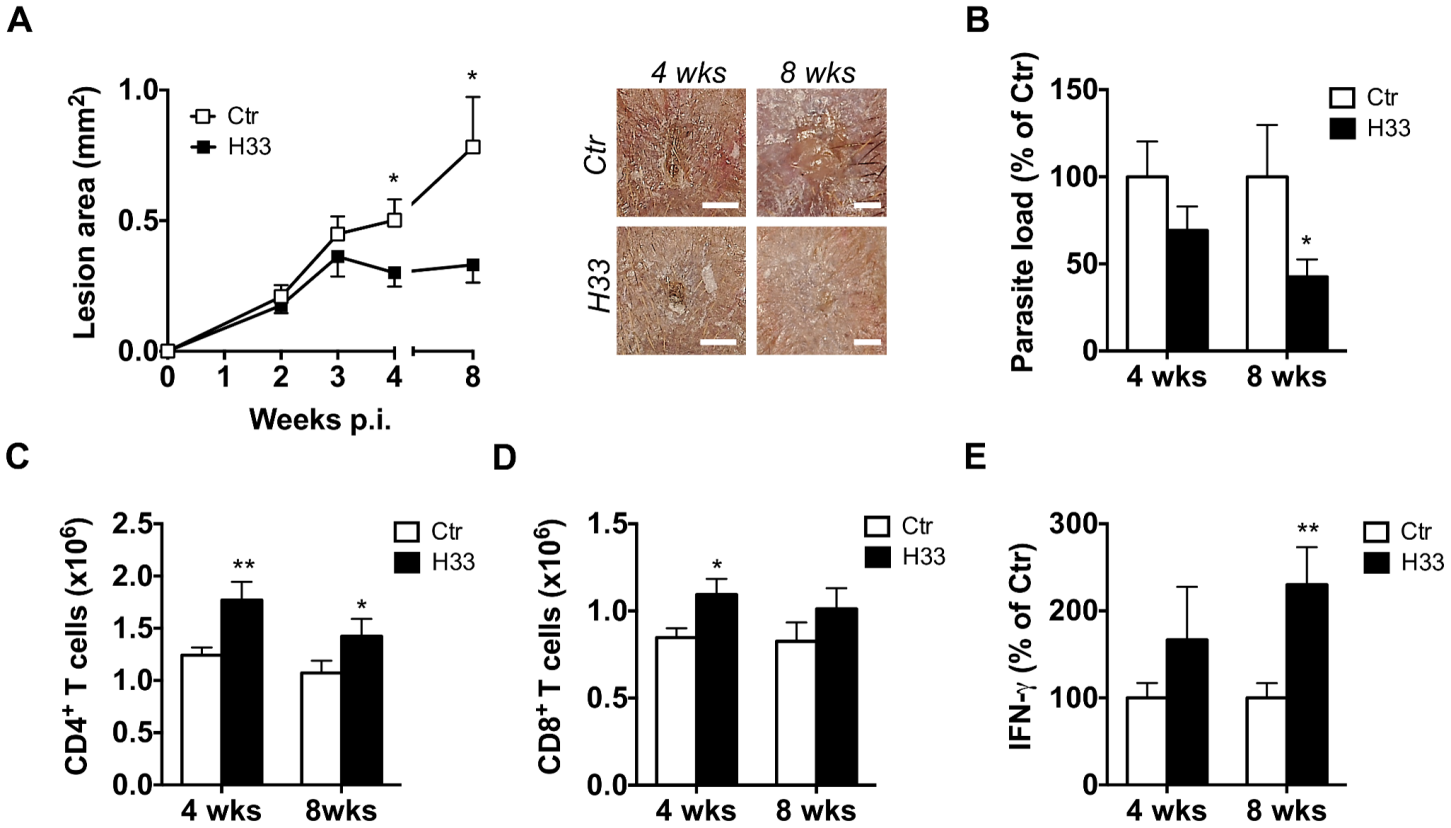
Blocking JAM-C improves the Th1 cell response and favours healing in C57BL/6 mice. (**A–E**) **Mice were inoculated with L. major in the ear dermis and treated with H33 or isotype control antibody for 3 weeks, twice a week.** (A) The area of the lesion was monitored weekly and representative pictures of ear lesions are shown at 4 and 8 weeks p.i. Scale bars, 0.5 mm. Data represent the mean ± SEM of twenty mice per group pooled from two separate experiments for the time point 4 weeks; and fifteen mice per group pooled from two separate experiments for the time point 8 weeks. (B) The parasite burden in infected ears was measured by LDA 4 and 8 weeks p.i. Data are expressed as a percentage of the mean of the control group ± SEM of mice from panel A. (C–D) The number of draining lymph node CD4^+^ (C) and CD8^+^ (D) T cells analyzed by flow cytometry 4 and 8 weeks p.i. Data represent the mean ± SEM of mice from panel A. (E) Draining lymph node cells were restimulated for 72 hrs with UV-irradiated *L. major* and the secreted IFN-γ was measured. Data are expressed as a percentage of the mean of the control group ± SEM of mice from panel A. Data were analyzed by the unpaired Student's t test with *:p<0.05 and **: p<0.01. For panels B and E, raw data from one experiment are also provided in S8 Figure.

The increased DC migration to the draining lymph node in mice treated with H33 raised the question of an eventual effect on the subsequent T cell response and disease outcome. As previously reported, the induction of the T cell response starts between the second and third week after infection [10]. Therefore, mice were infected with *L. major* and treated with H33 for 3 weeks in order to boost DC migration and T cell activation. The disease was followed weekly by measuring the area of the lesions, and we assessed the *L. major* specific T cell response together with the parasite burden 4 weeks and 8 weeks post infection (p.i.). In C57BL/6 mice, we found smaller lesions in H33-treated compared to control animals at both time points (Fig. 4A). Moreover, the reduction of the lesion area between the groups correlated with the decrease of the parasite load (Fig. 4B and S8 Figure). These results were in line with the increased numbers of CD4^+^ and CD8^+^ T cells observed (Fig. 4C and D). More importantly, draining lymph nodes T cells restimulated with UV-irradiated *L. major* produced significantly higher levels of IFN-γ at 8 weeks post infection, which accounts for the reduced lesion size and parasite load observed (Fig. 4E and S8 Figure). Taken together, these data suggest that H33 increases DC migration and therefore indirectly boosts the *L. major* specific IFN-γ-dominated Th1 cell response, resulting in a reduced severity of the disease.

### Blocking JAM-C boosts the Th2 cell response and worsens the disease in BALB/c mice

**Figure 5:**
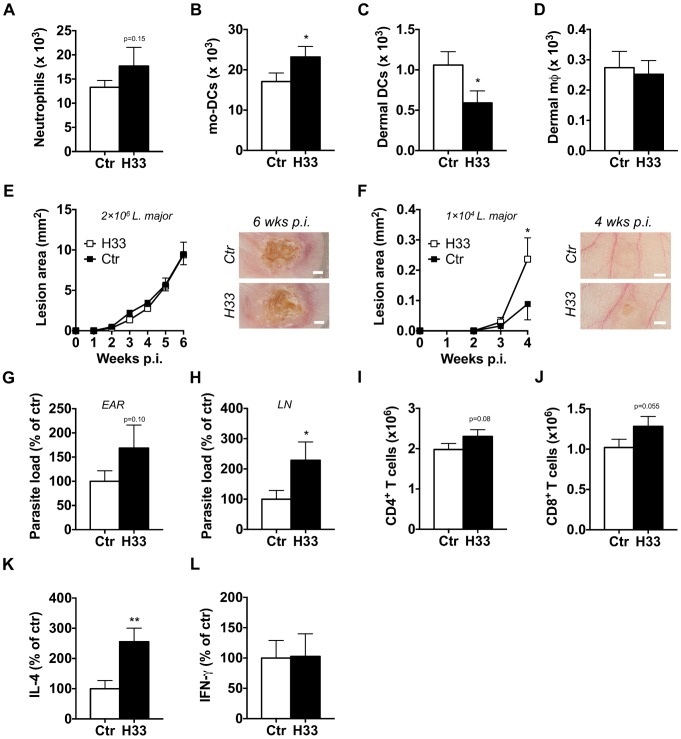
Blocking JAM-C boosts the Th2 cell response and worsens the disease in BALB/c mice. The number of emigrating neutrophils (A), mo-DCs (B), dermal DCs (C) and dermal mφ (D) was measured in the ears of H33-treated (H33, black bar) versus isotype control-treated mice (Ctr, white bars) 24 hours post *L. major* infection. Data represent the mean ± SEM of twelve mice per group pooled from 2 separate experiments, and were analyzed by the unpaired Student's t test with *: p<0.05. (E) Mice were inoculated with 2×10^6^ stationary phase *L. major* promastigotes in the ear dermis and treated with H33 or control antibody for 3 weeks. The area of the lesion was monitored weekly for 6 weeks. Representative ear pictures are shown. Scale bars, 1 mm. Data represent the mean ± SEM of twenty mice per group pooled from two separate experiments. (F–L) Mice were inoculated with 1×10^4^ stationary phase *L. major* promastigotes in the ear dermis and treated with H33 or control antibody for 3 weeks. The area of the lesion was monitored weekly for 4 weeks. Representative ear pictures are shown. Scale bars, 0.5 mm. Data represent the mean ± SEM of ten mice per group pooled from two separate experiments. (G–H) The parasite burden in infected ears (G) and draining lymph nodes (H) were measured by LDA. Data are expressed as a percentage of the mean of the control group ± SEM of mice from panel F. (I–J) The number of CD4^+^ (I) and CD8^+^ (J) T cells were measured. Data represent the mean ± SEM of mice from panel F. (K–L) Draining lymph nodes cells were restimulated with UV-irradiated *L. major* for 72 hours, and the IL-4 (K) and IFN-γ (L) produced were measured. Data are expressed as a percentage of the mean of the control group ± SEM of mice from panel F. Data were analyzed by the unpaired Student's t test with *:p<0.05 and **: p<0.01. For panels expressing results as a percentage of the mean of the control, raw data of one experiment are provided in S9 Figure.

Contrary to the C57BL/6 background, BALB/c mice develop a Th2 response promoting susceptibility rather than resistance to *L. major* infection [3]. Therefore, we investigated the effect of JAM-C blockade on leukocyte migration and disease outcome in susceptible BALB/c animals. After 24 hours of infection, we found increased numbers of neutrophils, and mo-DCs recruited to the site of infection in H33-treated BALB/c mice as compared to isotype control-treated mice (Fig. 5A and B). Moreover, we observed a decreased number of dermal DCs while unchanged numbers of dermal macrophages (Fig. 5C and D). These results showed that H33 influences leukocyte migration in a similar manner in BALB/c than in C57BL/6 mice. We next wanted to assess whether this increased leukocyte migration could change the dominance of the Th2 response over the Th1 response along the course of the disease. To this end, BALB/c mice were injected with the same dose of *L. major* used with C57BL/6 mice. We did not find any change in the disease outcome with H33, most likely as a result of an exaggerated Th2 polarization with high parasite doses (Fig. 5E). Therefore, we designed a new experiment with 200 fold less parasites inoculated. Strikingly, we now observed higher lesions in H33-treated animals (Fig. 5F). This correlated with increased parasite loads in ears and draining lymph nodes (Fig. 5G and H), while parasites were undetectable in spleens (S9 Figure). The number of T cells was also augmented in draining lymph nodes (Fig. 5I and J) and they secreted higher levels of IL-4 upon restimulation with UV-irradiated *L. major* (Fig. 5K). The production of IFN-γ was however unchanged (Fig. 5L). Altogether, these results show that the increased DC migration boosts the polarized Th2 immune response without changing the type of the T helper cell response.

## Discussion

In this study we investigated the involvement of JAM-C in the immune response against *L. major*. We first observed a decreased cell surface expression of endothelial JAM-C that corroborated with the strong accumulation of leukocytes at the site of *L. major* infection. We therefore postulated that JAM-C downregulation would render the endothelial junctions more permeable for inflammatory cells or fluids.

Previous findings reported that JAM-C mainly stabilizes cell junctions through trans-heterophilic, high affinity, low turnover interactions with its main partner JAM-B, while homophilic JAM-C-JAM-C interactions are weaker and occur with rapid dynamics [20]. The function of JAM-C in regulating endothelial permeability has been addressed by *in vivo* and *in vitro* studies using different approaches. *In vitro*, we have previously reported that CHO cells transfected with JAM-C exhibit an increased barrier function while MDCK cells transfected with JAM-C present increased paracellular permeability [22], [30]. When HUVEC cells were stimulated with the permeability factors VEGF or thrombin, JAM-C redistributed rapidly into cell-cell contacts and permeability was augmented [31], [32]. Overexpression of JAM-C in vitro also renders endothelial cells more permeable, probably due to the association in cis with the integrin αvβ3 [32]. These findings strongly suggest that the integrity of the endothelium is the result of a finely regulated ratio of junctional molecules. Moreover, one should also consider that overexpression of JAM-C in such *in vitro* systems may interfere with the biogenesis of endogenous junctional proteins with unpredictable consequences for the barrier function [33]. More recently, Chavakis and coworkers addressed the permeability question by using wild type mice treated with soluble recombinant JAM-C in a histamine-mediated vascular permeability model [34]. They reported that soluble JAM-C reduces vascular permeability in this particular model. It is worth noting that soluble JAM-C binds to JAM-C but can also engage strong interactions with JAM-B, or with other unknown ligands. Therefore, the effect of soluble JAM-C may be the sum of several interactions, making interpretation of these results difficult.

**Figure 6:**
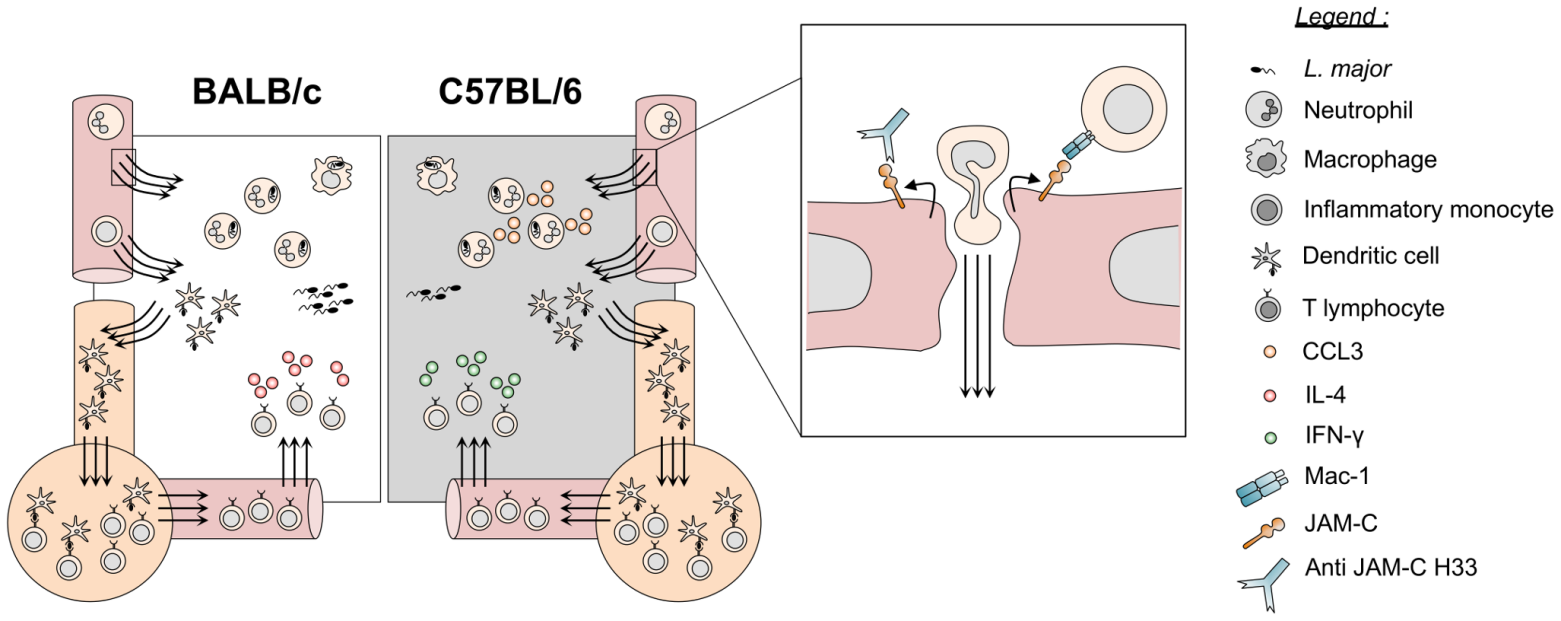
Blocking JAM-C enhances DC migration and boosts the immune responses to *L. major* infection. By removing JAM-C out of functions, H33 increases adhesion of leukocytes and potentiates vascular permeability and cell migration of leukocytes after *L. major* infection. Increased numbers of recruited neutrophils result in higher levels of the chemokine CCL3 attracting monocytes and mo-DCs in C57BL/6 mice. The number of migratory DCs to lymph nodes increases, and the subsequent T cell response is mounted more efficiently. Resistant C57BL/6 mice develop a higher IFN-γ-dominated Th1 response while susceptible BALB/c mice develop a stronger IL-4-dominated Th2 response. This has a significant healing effect in resistant animals whereas susceptible mice display an exacerbated disease.

To specifically address the function of JAM-C in vascular permeability *in vivo*, we used the multi-faceted H33 antibody that blocks JAM-C-JAM-B interactions and redistributes JAM-C out of tight junctions [20]. In our model, we were able to confirm that H33 removes JAM-C out from endothelial cell junctions. More importantly, this study showed for the first time that JAM-C blockade and redistribution with H33 increases vascular permeability by 15% after *L. major* inoculation in the skin. Conversely, we did not observe increased vascular permeability after administration of H33 in the steady state. This increase is substantial, as vascular permeability in inflammation is an optimized process [35]. Moreover, the absence of the H33 effect on vascular permeability in normal homeostasis is not surprising as many other different junctional molecules can still ensure vascular integrity in absence of inflammatory signals [36]. In line with this observation, H33 treatment also does not increase leukocyte migration from the blood to the tissue in absence of pathogen-mediated, inflammatory signals. However, after *L. major* infection, the number of leukocytes that migrate to the inflamed tissue increased significantly in mice treated with H33. As recent findings showed that VE-cadherin controls permeability and transmigration independently [37], our data with H33 may be in part the result of increased vascular permeability or the redistribution of JAM-C away from junctions as well. Redistribution of JAM-C on the apical side of the lumen makes it available for interactions with Mac-1 found on neutrophils and monocytes [20]. Accumulation of more adherent leukocytes on the luminal side of vessels could then increase the number of transmigrating cells. Therefore, H33 may increase leukocyte adhesion to the inflamed endothelium in addition to promoting vascular permeability in the context of *L. major* infection (Fig. 6).

In the *L. major* mouse model of cutaneous leishmaniasis, the kinetics of leukocyte recruitment, their specific function, and the crosstalk between the different subsets of cells have been and are still under investigation. It is now well accepted that neutrophils are the first cells recruited within hours to the infected tissue [10], [27], [38], [39]. However, some discrepancies still exist concerning their immunoregulatory function, which may depend on the mode of parasite transmission and the number of pathogens inoculated. Indeed, in the *Leishmania* resistant C57BL/6 mice, Sacks and coworkers used the monocyte and neutrophil depleting RB8-6C5 antibody and a natural sand-fly transmission of *L. major* to show that depletion of these cells promotes rather than compromises host resistance [38]. More recently, they showed that efferocytosis of infected neutrophils by DCs decreases their activation and antigen presenting cell function, therefore dampening the protective pro-inflammatory response [27]. On the other hand, other reports using needle inoculation of high parasite doses like our study, have demonstrated a transient protective role for neutrophils in C57BL/6 mice [40]–[42]. These studies assessed the role of neutrophils mostly by depletion, mediated by the more neutrophil-specific antibody NIMP-R14 [39] or by the anti-Gr1 RB6-8C5 antibody recognizing inflammatory monocytes and neutrophils. All these neutrophil depletion studies resulted in transient increased lesion size and parasite loads. It is worth noting that RB6-8C5, the antibody used in most of the studies, not only depletes neutrophils but also inflammatory monocytes, illustrating the importance of neutrophils and inflammatory monocytes in promoting resistance to infection. The contribution of monocytes and mo-DCs has been further investigated with the use of chemokine receptor CCR2 knock-out mice in the resistant C57BL/6 background. In these mice, monocytes do not leave the bone marrow, resulting in a deficiency of monocytes in the blood circulation [43]. The recruitment of inflammatory mo-DCs in the lymph node following *L. major* infection is therefore completely impaired, which dampens the Th1 cell response [11]. Subsequently, the CCR2 deficiency results in a non-healing phenotype similar to that observed in susceptible mice [12]. Our report is in line with these studies, as treatment with the antibody H33 increased the recruitment of neutrophils, inflammatory monocytes and mo-DCs, and thereby improving the Th1 immune response and the clinical outcome in C57BL/6 mice. In addition, Tacchini-Cottier and coworkers [26] emphasized the contribution of neutrophils in the recruitment of mo-DCs to the site of *L. major* infection through the secretion of the chemokine CCL3. In line with this finding, we observed a significant increase in the production of CCL3 within ears of H33 treated mice at time points where neutrophils are massively recruited to the site of infection. Therefore, we suggest that, by increasing the numbers of neutrophils recruited, H33 could indirectly increase the amount of CCL3 produced *in situ*. This additional mechanism may also contribute to further enhance the extravasation of mo-DCs in the ears of H33 treated C57BL/6 mice (Fig. 6).

We also report that H33 increases the number of DCs leaving the ear dermis to the draining lymph node. This may be the direct consequence of JAM-C blocking at lymphatic endothelial cell junctions, which would facilitate DCs transendothelial migration. Alternatively, it could be the indirect consequence of the higher number of DCs recruited to the site of inflammation that migrate to the lymph node as a consequence. The later hypothesis is more likely since DCs preferentially migrate in an integrin-independant manner through initial lymphatic capillaries by seeking pre-existing flaps between the oak leaf-shaped lymphatic endothelial cells [44], [45]. It is worth noting that JAM-C is also well expressed by lymphatic sinuses from lymph nodes, which may influence T cell surveillance of DCs in the lymph nodes, and therefore T cell activation after H33 treatment. However, we had already demonstrated that the ability of DCs to prime T cells *in vivo* in JAM-C deficient animals is unchanged [46]. In lymph nodes, JAM-C is also expressed by high endothelial venules, and we cannot exclude an effect of H33 on T cell entry into lymph nodes.

Finally, we used the properties of H33 treatment on vascular permeability and innate cell migration to assess the consequences on the clinical outcome. In C57BL/6 mice, the higher numbers of neutrophils or monocyte-derived cells recruited rapidly after infection may increase the early innate parasite killing. However, we observed no difference in the parasite load 48 hours after infection. This is likely due to the absence of the IFN-γ-dominated Th1 response that leads to activation of phagocytes and parasite killing at this early time point [3]. Moreover, the increased vascular permeability at the site of infection may have influenced the dissemination of the parasite to peripheral organs early after infection. But we did not found any change in the draining lymph nodes, while the parasite was undetectable in spleen. Strikingly, we found that H33 was able to boost the adaptive immune response in both susceptible and resistant mice by increasing DC migration, without changing the T cell polarization. This report is the first one to demonstrate that immune responses to pathogen infections can be finely-tuned by manipulating a single adhesion molecule, and in particular JAM-C. For instance, deletion of P- or E-selectin does not impact the immune response to *L. major* infection in a mixed 129/C57BL/6 background [47]. Finally, our findings in BALB/c mice confirm that susceptibility does not result from an overall lack of leukocyte migration to the site of infection, but rather from a genetic defect in redirecting the T cell response [3].

## Materials and Methods

### Ethics statement

All animal procedures were performed in accordance with the Institutional Ethical Committee of Animal Care in Geneva, Switzerland. The protocol has been approved by the Ethics and Federal Veterinary office regulations of the state of Geneva. Our laboratory has the authorization number 1005-3753.1.

### Mice and parasites

Female C57BL/6J and BALB/c mice were purchased from Charles River (Lyon, France). Mice were bred in the P2 animal facility at the CMU, and used between 6–8 weeks of age. *Leishmania major* LV39 (MRHO/Sv/59/P Strain) were used. In all experiments, C57BL/6 mice were infected in the ear dermis with 2×10^6^ stationary phase *L. major* promastigotes in a volume of 10 µL. The disease outcome in BALB/c was followed after infection with 2×10^6^ and 1×10^4^ stationary phase *L. major* promastigotes in a volume of 10 µL.

### Flow cytometry analysis of ear endothelial cells

The ventral and dorsal sheets of mouse ears were split with forceps, and digested with 3 mg/mL collagenase type IV (Invitrogen) and 1 mg/mL DNAse type I (Sigma Aldrich) for 45 minutes at 37°C, filtered through a 70 µm gauge strainer (Becton Dickinson), and the cells labelled for FACS analysis. Fc receptors were blocked with the monoclonal antibody (mAb) 2.4G2 (Becton Dickinson). Cells were stained with the following reagents: Alexa Fluor 488-conjugated anti-mouse podoplanin (clone 8.1.1), PE-conjugated anti-mouse CD31 (clone 390), PE-Cy7-conjugated anti-mouse CD45 (clone 30-F11), all from affimetrix eBioscience. JAM-C was labelled with an affinity purified polyclonal anti-mouse JAM-C antibody raised in rabbit [31], while affinity purified rabbit IgG (Sigma) was used as a control. The secondary antibody used was an Alexa Fluor 647-conjugated anti-rabbit antibody (Jackson ImmunoResearch). Cells were analyzed with a Gallios FACS machine (Beckman Coulter) and the data were processed with Kaluza software (Beckman Coulters).

### Leukocyte emigration for ear skin explants and FACS analysis

Mice were injected i.p. with the rat IgG2a anti-mouse JAM-C H33 or the rat IgG2a isotype control 2A3 (BioXCell), 200 µg/mice, 2 hours before inoculation of *L. major* in the ear dermis. Twenty-four hours post infection, mice were sacrificed and ears explanted. The ventral and dorsal sheets of the ears were separated with forceps, and transferred overnight in twelve well plates filled with RPMI-1640 medium supplemented with 10% fetal calf serum and antibiotics at 37°C. Over this period of time, the leukocytes that have been recruited to the infected ears spontaneously emigrated from the explants. Emigrated cells were then counted with a hemocytometer, and stained for FACS analysis. Fc receptors were blocked with the mAb 2.4G2. Cells were stained with the following reagents: Alexa Fluor 488-conjugated anti-mouse Ly6C (clone HK1.4, Biolegend), PercP-Cy5.5-conjugated anti-mouse Ly6G (clone 1A8, Biolegend), PE-Cy7-conjugated anti-mouse CD11b (clone M1/70, Biolegend), APC-Cy7-conjugated anti-mouse CD11c (clone N418, Biolegend), and efluor 450-conjugated anti-mouse IA/IE (clone M5/114.15.2, eBiosciences). Cells were analyzed with a Gallios FACS machine (Beckman Coulter) and data processed with Kaluza software (Beckman Coulters). The number of cells per population was calculated by multiplying the total number of emigrating cells with the percentage of cells of interest.

### FACS analysis of leukocyte populations in steady state

Mice were injected i.p. with the mAb H33 or the control mAb 2A3 (200 µg/mice). Mice were then sacrificed 24 hours after treatment to collect ears, blood and femurs. Ears were processed as described above. Femurs were flushed to extract bone marrow cells. Red blood cells from blood and bone marrow samples were lysed with Ammonium-Chloride-Potassium (ACK) lysis buffer. A fraction of each sample was used for FACS staining using BD Trucount tubes according to the manufacturer's instructions. Fc receptors were blocked with the mAb 2.4G2. Bone marrow cells were stained with the following reagents: Alexa Fluor 488-conjugated anti-mouse Ly6C (clone HK1.4, Biolegend), PE-conjugated anti-mouse CD115 (clone AFS98, eBiosciences), PercP-Cy5.5-conjugated anti-mouse Ly6G (clone 1A8, Biolegend), PE-Cy7-conjugated anti-mouse F4/80 (clone BM8, Biolegend), APC-conjugated anti-mouse CD11c (clone HL3, BD), APC-Cy7-conjugated anti-mouse TCR_β_ (clone H57-597, Biolegend), efluor 450-conjugated anti-mouse CD11b (clone M1/70, eBiosciences), Brilliant Violet 785-conjugated anti-mouse CD8α (clone 53-6.7, Biolegend). Blood cells were stained with the following reagents: Alexa Fluor 488-conjugated anti-mouse Ly6C (clone HK1.4, Biolegend), PE-conjugated anti-mouse CD115 (clone AFS98), PercP-Cy5.5-conjugated anti-mouse Ly6G (clone 1A8, Biolegend), PE-Cy7-conjugated anti-mouse CD4 (clone RM4-5, Biolegend), APC-conjugated anti-mouse NK1.1 (clone PK136, Biolegend), APC-Cy7-conjugated anti-mouse CD19 (clone 6D5, Biolegend), efluor 450-conjugated anti-mouse CD11b (clone M1/70, eBiosciences), Brilliant Violet 785-conjugated anti-mouse CD8α (clone 53-6.7, Biolegend). Cells were analyzed with a Gallios FACS machine (Beckman Coulter) and the data were processed with Kaluza software (Beckman Coulter). The number of cells per population was calculated by multiplying the total number of cells with the percentage of cells of interest. The total number of cells was calculated using the number of Trucount beads analyzed by the flow cytometer.

### FITC painting experiments

Mice were injected i.p. with the mAb H33 or the control mAb 2A3 (200 µg/mice) 2 hours before FITC painting of mice ears. FITC (Sigma) was used at 5 mg/mL and dissolved in aceton: dibutyl phthalate (1∶1, v∶v). Twenty microliters were applied to each side of the ear. Eighteen hours after painting, the ear draining lymph node was harvested and digested with 3 mg/mL collagenase type IV (Invitrogen) and 1 mg/mL DNAse type I (Sigma) for 45′ at 37°C, and filtered through a 70 µm gauge strainer (Becton Dickinson). The cells were counted with a hemocytometer, and labelled for FACS analysis. Fc receptors were blocked with the mAb 2.4G2. Cells were stained with the following reagents: APC-Cy7-conjugated anti-mouse CD11c, and efluor 450-conjugated anti-mouse IA/IE. Cells were analyzed with a Gallios FACS machine (Beckman Coulters) and data processed with Kaluza software (Beckman Coulters). The number of FITC^+^ migratory DCs was calculated by multiplicating the total number of lymph node cells with the percentage of IA/IE^high^ CD11c^+^ FITC^+^ DCs.

### Immunofluorescence microscopy

Mice were injected i.p. with the mAb H33 or the control mAb 2A3 (200 µg/mice). Twenty-four hours after injection, ears were embedded in Tissue-Tek OCK compound, frozen at −80°C, then cut (5 µm) with a cryostat. Fresh ear sections were fixed in cold acetone for 5 minutes, rehydrated in PBS for 10 minutes, and blocked with 10% normal donkey serum. CD31 was detected with an Alexa Fluor 647-conjugated rat anti mouse CD31 (clone GC51, home made), while JAM-C was detected with a polyclonal anti-mouse JAM-C antibody raised in rabbit [31] followed by an Alexa Fluor 488-conjugated donkey anti-rabbit antibody (Jackson ImmunoResearch). We used rabbit IgG as control for JAM-C staining. Cell nucleus was stained with DAPI and slides were mounted with mowiol mounting medium. Labelled ear sections were visualized with a Nikon A1R confocal microscope and the NIS Elements AR software. All images were acquired with a 100× objective. The maximal intensity projection image of the z-stack is shown. The images were analyzed with Image J. The distribution profile of JAM-C was ploted along the minor axis of the cells.

### Vascular permeability assay

Mice were treated i.p. with the mAb H33 or the control mAb 2A3 (200 µg/mice) 2 hours before 100 µL of Evans blue (12 mg/mL) was injected i.v. and *L. major* inoculated i.d. in the ear. Five hours after infection, mice were killed, and the permeability of Evans blue in the ear documented by picturing each ear. Ears were then cut, weighted, split into dorsal and ventral sheets, and finally transferred into formamide for 2 days at room temperature to extract the Evans blue dye. The absorbance of the samples was measured at 620 nm (Ledetect 96, Labexim) and normalized to the weight of tissue.

### CCL3 level in ear following *L. major* infection

Mice were injected i.p. with H33 or the control mAb 2A3 (200 µg/mice) 2 hours before *L. major* inoculation in the ear dermis. Eight or 24 hours after infection, ears were homogenized on ice in a protease inhibitor cocktail (Sigma Aldrich, P8340) using a polytron as tissue homogenizer. The expression of the chemokine CCL3 were measured in tissue homogenates with the BD CBA mouse Flex Set kit according to the manufacturer instructions. Beads were analyzed on a Cyan (Beckman Coulters) flow cytometer and data processed with the FCAP array software (Becton Dickinson).

### T cell response in the draining lymph node and cytokine detection

The ear draining lymph nodes were digested with 3 mg/mL collagenase type IV (Invitrogen) and 1 mg/mL DNAse type I (Sigma) for 45′ at 37°C, and filtered through a 70 µm gauge strainer (Becton Dickinson). The cells were counted with a hemacytometer and labelled for FACS analysis. Fc receptors were blocked with the mAb 2.4G2. Cells were stained for cell surface antigens with the following reagents: FITC-conjugated anti-mouse TCRβ (clone H57-597, eBioscience), Brilliant Violet 421-conjugated anti-mouse CD8α (clone 53-6.7, Biolegend), Brilliant Violet 785-conjugated anti-mouse CD4 (clone RM4-5, Biolegend). Cells were analyzed with a Gallios FACS machine (Beckman Coulters) and the data were processed with Kaluza software (Beckman Coulters). The number of cells per population was calculated by multiplying the total number of lymph nodes cells with the percentage of cells of interest. For T cell restimulation, draining lymph nodes cells were incubated at 37°C under 5% CO_2_ for 72 hours in the presence of UV-irradiated *L. major* (ratio 5∶1, cell∶parasite). Supernatant were collected and the levels of IL-4 and IFN-γ were measured by ELISA (eBioscience) or CBA (Becton Dickinson) according to the manufacturer instructions.

### Lesion area measurement and parasite load

Mice were injected i.p. with the mAb H33 or the control mAb 2A3 (200 µg/mice) 2 hours before inoculation of *L. major* in the ear dermis. Injections of mAbs (100 µg/mice) were repeated twice a week for twenty-one days. The evolution of the lesion was documented weekly with a picture of each ear, as well as the picture of a 1 cm scale. The camera was fixed on a support for the scale to be unchanged from one picture to the other. The pictures were analyzed with ImageJ software. Briefly, the picture of the 1 cm scale provides the number of pixels per 1 cm unit. Each lesion was then defined manually with the software, and the precise lesion area calculated using the number of pixels in the selected area. For parasite burden, the infected ears were explanted, weighted, and separated into two halves. Ear leaflets were enzymatically digested before tissue dissociation with a gentleMACS Octo Dissociator (Miltenyi Biotech). Ears homogenates, lymph nodes or spleens cells were serially diluted, and the parasite load estimated by limiting dilution assay as described [48].

### Statistical analysis

Data were analyzed with the GraphPad Prism statistics software. We used the Student's t-test for unpaired data for all experiments.

## Supporting Information

Figure S1 **Leukocytes emigrating to the site of *L. major* infection do not express JAM-C.** The expression of JAM-C by leukocytes emigrated from *L. major* infected ears was measured 24 hours post infection. CD11b^+^ Ly6C^+^ Ly6G^+^ represent neutrophils, CD11b^+^ Ly6C^+^ Ly6G^−^ CD11c^−^ IA^−^ are monocytes, CD11b^+^ Ly6C^+^ Ly6G^−^ CD11c^+^ IA^+^ are mo-DCs, CD11b^+^ Ly6C^−^ Ly6G^−^ CD11c^low^ IA^+^ are dermal mφ, and CD11b^+^ Ly6C^−^ Ly6G^−^ CD11c^high^ IA^+^ are dermal DCs. A representative histogram overlay of JAM-C expression is shown for each population, with JAM-C staining (black line), and isotype control staining (grey line). Data are representative of two separate experiments.
(TIFF)

Figure S2 **JAM-C expression in ear endothelial cells does not decrease 24 hours after saline injection (A) JAM-C levels in endothelial cells populations of mouse ear.** Ears were enzymatically digested and stained for FACS analysis. CD45^−^ CD31^+^ gp38^−^ cells represent blood endothelial cells (BECs), whereas CD45^−^ CD31^+^ gp38^+^ cells are lymphatic endothelial cells (LECs). For each population a representative histogram overlay is shown with JAM-C in endothelial cells from naïve ears (white filled), JAM-C in endothelial cells from saline injected ears (black filled), and the isotype control staining (grey filled). (B) The MFI of JAM-C in naïve mouse ears (white bars) versus saline injected mouse ears (black bars) was measured in BECs and LECs. The Y-axis scale represents MFI normalized to the mean MFI of naïve ears. Data represent the mean ± SEM of five mice per group pooled from two separate experiments.
(TIFF)

Figure S3 **Control staining for JAM-C in ear endothelial cells.** Ear sections were stained for Rabbit IgG control (green), CD31 (red). Nucleus was stained with DAPI (blue). Scale bars, 10 µm. This supporting information is related to Fig. 1D.
(TIFF)

Figure S4 **Blocking JAM-C does not result in leukocyte emigration to tissue in the steady state.** The number of neutrophils (A), monocytes (B), mo-DCs (C), dermal mφ (D), and dermal DCs (E) emigrating from ears was measured in H33-treated (H33, black bar) versus isotype control-treated mice (Ctr, white bars) 24 hours after antibody administration. Data represent the mean ± SEM of fifteen mice per group pooled from 3 separate experiments, and were analyzed by the unpaired Student's t test.
(TIFF)

Figure S5 **Blocking JAM-C in the steady state does neither increase hematopoiesis nor leukocyte migration from bone marrow to the blood.** Naïve C57BL/6 mice were treated with H33 or isotype control antibody for 24 hours. The number of neutrophils (A), monocytes (B), DCs (C), T cells (D), eosinophils (E), and macrophages (F) from the bone marrow (BM); and B cells (G), CD4^+^ T cells (H), CD8^+^ T cells (I), neutrophils (J), monocytes (K), and NK cells (L) from blood in H33-treated (black bar) versus isotype control-treated mice (white bars) is shown. Data represent the mean ± SEM of five mice per group, and were analyzed by the unpaired Student's t test. Data are representative of three separate experiments.
(TIFF)

Figure S6 **H33 antibody does neither decrease the parasite burden in infected ears, nor increase parasite dissemination to lymph nodes 48 hours p.i. (Raw data of** **Fig. 2** **).** The parasite burden in infected ears (A) and draining lymph nodes (B) were measured 48 hours p.i. by LDA. Data represent the mean ± SEM of five mice per group from one representative experiment, and were analyzed by the unpaired Student's t test. These supporting informations are related to Fig. 2H and I.
(TIFF)

Figure S7 **Blocking JAM-C increases the number of DCs migrating to the draining lymph node (Raw data of** **Fig. 3** **).** The ear draining lymph nodes were harvested and stained for FACS analysis 18 hours after FITC-painting. The number of IA^high^ CD11c^+^ FITC^+^ migratory DCs was counted. Data represent the mean ± SEM of six mice per group, and were analyzed by the unpaired Student's t test with *: p<0.05. This supporting information is related to Fig. 3B.
(TIFF)

Figure S8 **Blocking JAM-C improves the Th1 cell response and favours healing in C57BL/6 mice (Raw data of** **Fig. 4** **).** Mice were inoculated with *L. major* in the ear dermis and treated with H33 or the isotype control antibody for 3 weeks, twice a week. (A) The parasite burden in infected ears was measured by LDA 4 and 8 weeks p.i. Data represent mean ± SEM of ten mice per group for both time points. (B) Draining lymph node cells were restimulated for 72 hrs with UV-irradiated *L. major* and the secreted IFN-γ was measured. Data represent the mean ± SEM of mice from panel A. Data were analyzed by the unpaired Student's t test with *:p<0.05. These supporting informations are related to Fig. 4B and E.
(TIFF)

Figure S9 **Blocking JAM-C boosts the Th2 cell response and worsens the disease in BALB/c mice (Raw data of** **Fig. 5** **).** (A–C) Mice were inoculated with 1×10^4^ stationary phase *L. major* promastigotes in the ear dermis and treated with H33 or control antibody for 3 weeks. The parasite burden in infected ears (A), draining lymph nodes (B), and spleens (C) were measured by LDA. (D–E) Draining lymph nodes cells were restimulated with UV-irradiated *L. major* for 72 hours, and the IL-4 (D) and IFN-γ (E) produced were measured. Data represent the mean ± SEM of 5 mice per group. Data were analyzed by the unpaired Student's t test with *:p<0.05, ***: p<0.001. n.d. not-detectable. These supporting informations are related to Fig. 5G, H, K and L.
(TIFF)

## References

[1] Murray HW, Berman JD, Davies CR, Saravia NG (2005) Advances in leishmaniasis. The Lancet 366: 1561–1577. 10.1016/S0140-6736(05)67629-5 16257344

[2] Kaye P, Scott P (2011) Leishmaniasis: complexity at the host-pathogen interface. Nat Rev Microbiol 9: 604–615. 21747391 10.1038/nrmicro2608

[3] Sacks D, Noben-Trauth N (2002) The immunology of susceptibility and resistance to Leishmania major in mice. Nat Rev Immunol 2: 845–858. 12415308 10.1038/nri933

[4] Heinzel F, Sadick M, Mutha S, Locksley R (1991) Production of interferon-gamma, interleukin-2, interleukin-4, and interleukin-10 by CD4+ lymphocytes in vivo during healing and progressive murine leishmaniasis. PNAS 88: 7011–7015. 1908085 10.1073/pnas.88.16.7011 PMC52223

[5] Wang ZE, Reiner LR, Zheng S, Dalton DK, Lockley RM (1994) CD4 + Effector Cells Default to the Th2 Pathway in Interferon y-deficient Mice Infected with Leishmania major. J Exp Med 179: 1367–1371. 7908325 10.1084/jem.179.4.1367 PMC2191434

[6] Liew F, Millott S, Parkinson C, Palmer R, Moncada S (1990) Macrophages killing of Leishmania parasite in vivo is mediated by nitric oxide from L-arginine. J Immunol 144: 4794–4797. 2351828

[7] Tacchini-Cottier F, Weinkopff T, Launois P (2012) Does T Helper Differentiation Correlate with Resistance or Susceptibility to Infection with L. major? Some Insights From the Murine Model. Front Immunol 3: 32. 22566916 10.3389/fimmu.2012.00032 PMC3342012

[8] Ng LG, Hsu A, Mandell MA, Roediger B, Hoeller C, et al (2008) Migratory dermal dendritic cells act as rapid sensors of protozoan parasites. PLoS Pathog 4: e1000222. 19043558 10.1371/journal.ppat.1000222 PMC2583051

[9] Von Stebut E, Belkaid Y, Jakob T, Sacks D, Udey M (1998) Uptake of Leishmania major Amastigotes Results in Activation and Interleukin 12 Release from Murine Skin–derived Dendritic Cells: Implications for the Initiation of Anti-Leishmania Immunity. J Exp Med 188: 1547–1552. 9782133 10.1084/jem.188.8.1547 PMC2213412

[10] Leon B, Lopez-Bravo M, Ardavin C (2007) Monocyte-derived dendritic cells formed at the infection site control the induction of protective T helper 1 responses against Leishmania. Immunity 26: 519–531. 17412618 10.1016/j.immuni.2007.01.017

[11] De Trez C, Magez S, Akira S, Ryffel B, Carlier Y, et al (2009) iNOS-producing inflammatory dendritic cells constitute the major infected cell type during the chronic Leishmania major infection phase of C57BL/6 resistant mice. PLoS Pathog 5: e1000494. 19557162 10.1371/journal.ppat.1000494 PMC2695779

[12] Sato N, Ahuja SK, Quinones M, Kostecki V, Reddick RL, et al (2000) CC Chemokine Receptor (CCR)2 Is Required for Langerhans Cell Migration and Localization of T Helper Cell Type 1 (Th1)-inducing Dendritic Cells: Absence of CCR2 Shifts the Leishmania major–resistant Phenotype to a Susceptible State Dominated by Th2 Cytokines, B Cell Outgrowth, and Sustained Neutrophilic Inflammation. J Exp Med 192: 205–218. 10899907 10.1084/jem.192.2.205 PMC2193245

[13] Ley K, Laudanna C, Cybulsky MI, Nourshargh S (2007) Getting to the site of inflammation: the leukocyte adhesion cascade updated. Nat Rev Immunol 7: 678–689. 17717539 10.1038/nri2156

[14] Scheiermann C, Colom B, Meda P, Patel NS, Voisin MB, et al (2009) Junctional adhesion molecule-C mediates leukocyte infiltration in response to ischemia reperfusion injury. Arterioscler Thromb Vasc Biol 29: 1509–1515. 19574560 10.1161/ATVBAHA.109.187559 PMC2746810

[15] Liang T, Chiu H, Gurney A, Sidle A, Tumas D, et al (2002) Vascular Endothelial-Junctional Adhesion Molecule (VE-JAM)/JAM 2 Interacts with T, NK, and Dendritic Cells Through JAM 3. J Immunol 168: 1618–1626. 11823489 10.4049/jimmunol.168.4.1618

[16] Santoso S, Sachs UJH, Kroll H, Linder M, Ruf A, et al (2002) The Junctional Adhesion Molecule 3 (JAM-3) on Human Platelets is a Counterreceptor for the Leukocyte Integrin Mac-1. J Exp Med 196: 679–691. 12208882 10.1084/jem.20020267 PMC2194005

[17] Zen K, Babbin BA, Liu Y, Whelan JB, Nusrat A, et al (2004) JAM-C is a component of desmosomes and a ligand for CD11b/CD18-mediated neutrophil transepithelial migration. Mol Biol Cell 15: 3926–3937. 15194813 10.1091/mbc.E04-04-0317 PMC491847

[18] Morris AP, Tawil A, Berkova Z, Wible L, Smith CW, et al (2006) Junctional Adhesion Molecules (JAMs) are differentially expressed in fibroblasts and co-localize with ZO-1 to adherens-like junctions. Cell Commun Adhes 13: 233–247. 16916751 10.1080/15419060600877978

[19] Scheiermann C, Meda P, Aurrand-Lions M, Madani R, Yiangou Y, et al (2007) Expression and function of junctional adhesion molecule-C in myelinated peripheral nerves. Science 318: 1472–1475. 18048693 10.1126/science.1149276 PMC3299566

[20] Lamagna C, Meda P, Mandicourt G, Brown J, Gilbert RJ, et al (2005) Dual interaction of JAM-C with JAM-B and alpha(M)beta2 integrin: function in junctional complexes and leukocyte adhesion. Mol Biol Cell 16: 4992–5003. 16093349 10.1091/mbc.E05-04-0310 PMC1237098

[21] Cunningham SA, Rodriguez JM, Arrate MP, Tran TM, Brock TA (2002) JAM2 interacts with alpha4beta1. Facilitation by JAM3. J Biol Chem 277: 27589–27592. 12070135 10.1074/jbc.C200331200

[22] Aurrand-Lions M, Duncan L, Ballestrem C, Imhof BA (2001) JAM-2, a novel immunoglobulin superfamily molecule, expressed by endothelial and lymphatic cells. J Biol Chem 276: 2733–2741. 11053409 10.1074/jbc.M005458200

[23] Bradfield PF, Scheiermann C, Nourshargh S, Ody C, Luscinskas FW, et al (2007) JAM-C regulates unidirectional monocyte transendothelial migration in inflammation. Blood 110: 2545–2555. 17625065 10.1182/blood-2007-03-078733 PMC1988941

[24] Woodfin A, Voisin MB, Beyrau M, Colom B, Caille D, et al (2011) The junctional adhesion molecule JAM-C regulates polarized transendothelial migration of neutrophils in vivo. Nat Immunol 12: 761–769. 21706006 10.1038/ni.2062 PMC3145149

[25] Vestweber D (2012) Relevance of endothelial junctions in leukocyte extravasation and vascular permeability. Ann N Y Acad Sci 1257: 184–192. 22671605 10.1111/j.1749-6632.2012.06558.x

[26] Charmoy M, Brunner-Agten S, Aebischer D, Auderset F, Launois P, et al (2010) Neutrophil-derived CCL3 is essential for the rapid recruitment of dendritic cells to the site of Leishmania major inoculation in resistant mice. PLoS Pathog 6: e1000755. 20140197 10.1371/journal.ppat.1000755 PMC2816696

[27] Ribeiro-Gomes FL, Peters NC, Debrabant A, Sacks DL (2012) Efficient capture of infected neutrophils by dendritic cells in the skin inhibits the early anti-leishmania response. PLoS Pathog 8: e1002536. 22359507 10.1371/journal.ppat.1002536 PMC3280984

[28] Miles AA, Miles EM (1952) Vascular reactions to histamine, histamine-liberator and leukotaxine in the skin of guinea-pigs. J Physiol 118: 228–257. 13000707 10.1113/jphysiol.1952.sp004789 PMC1392441

[29] Robbiani DF, Finch RA, Jager D, Muller WA, Sartorelli AC, et al (2000) The Leukotriene C4 Transporter MRP1 Regulates CCL19 (MIP-3b, ELC)–Dependent Mobilization of Dendritic Cells to Lymph Nodes. Cell 103: 757–768. 11114332 10.1016/s0092-8674(00)00179-3

[30] Aurrand-Lions M, Johnson-Leger C, Wong C, Du Pasquier L, Imhof B (2001) Heterogeneity of endothelial junctions is reflected by differential expression and specific subcellular localization of the three JAM family members. Blood 98: 3699–3707. 11739175 10.1182/blood.v98.13.3699

[31] Lamagna C, Hodivala-Dilke KM, Imhof BA, Aurrand-Lions M (2005) Antibody against Junctional Adhesion Molecule-C Inhibits Angiogenesis and Tumor Growth. Cancer Res 65: 5703–5710. 15994945 10.1158/0008-5472.CAN-04-4012

[32] Li X, Stankovic M, Lee BP, Aurrand-Lions M, Hahn CN, et al (2009) JAM-C induces endothelial cell permeability through its association and regulation of {beta}3 integrins. Arterioscler Thromb Vasc Biol 29: 1200–1206. 19461049 10.1161/ATVBAHA.109.189217

[33] Tenan M, Aurrand-Lions M, Widmer V, Alimenti A, Burkhardt K, et al (2010) Cooperative expression of junctional adhesion molecule-C and -B supports growth and invasion of glioma. Glia 58: 524–537. 19795504 10.1002/glia.20941

[34] Orlova VV, Economopoulou M, Lupu F, Santoso S, Chavakis T (2006) Junctional adhesion molecule-C regulates vascular endothelial permeability by modulating VE-cadherin-mediated cell-cell contacts. J Exp Med 203: 2703–2714. 17116731 10.1084/jem.20051730 PMC2118160

[35] Spindler V, Schlegel N, Waschke J (2010) Role of GTPases in control of microvascular permeability. Cardiovasc Res 87: 243–253. 20299335 10.1093/cvr/cvq086

[36] Goddard LM, Iruela-Arispe ML (2013) Cellular and molecular regulation of vascular permeability. Thromb Haemost 109: 407–415. 23389236 10.1160/TH12-09-0678 PMC3786592

[37] Wessel F, Winderlich M, Holm M, Frye M, Rivera-Galdos R, et al (2014) Leukocyte extravasation and vascular permeability are each controlled in vivo by different tyrosine residues of VE-cadherin. Nat Immunol 10.1038/ni.2824 24487320

[38] Peters NC, Egen JG, Secundino N, Debrabant A, Kimblin N, et al (2008) In Vivo Imaging Reveals an Essential Role for Neutrophils in Leishmaniasis Transmitted by Sand Flies. Science 321: 970–974. 18703742 10.1126/science.1159194 PMC2606057

[39] Tacchini-Cottier F, Zweifel C, Belkaid Y, Mukankundiye C, Vasei M, et al (2000) An Immunomodulatory Function for Neutrophils During the Induction of a CD4 + Th2 Response in BALB/c Mice Infected with Leishmania major. J Immunol 165: 2628–2636. 10946291 10.4049/jimmunol.165.5.2628

[40] Lima GM, Vallochi AL, Silva UR, Bevilacqua EM, Kiffer MM, et al (1998) The role of polymorphonuclear leukocytes in the resistance to cutaneous Leishmaniasis. Immunol Lett 64: 145–151. 9870666 10.1016/s0165-2478(98)00099-6

[41] Ribeiro-Gomes FL, Otero AC, Gomes NA, Moniz-de-Souza MCA, Finkelstein LC, et al (2004) Macrophage Interactions with Neutrophils Regulate Leishmania major Infection. J Immunol 172: 4454–4462. 15034061 10.4049/jimmunol.172.7.4454

[42] Chen L, Zhang ZH, Watanabe T, Yamashita T, Kobayakawa T, et al (2005) The involvement of neutrophils in the resistance to Leishmania major infection in susceptible but not in resistant mice. Parasitol Int 54: 109–118. 15866472 10.1016/j.parint.2005.02.001

[43] Serbina NV, Pamer EG (2006) Monocyte emigration from bone marrow during bacterial infection requires signals mediated by chemokine receptor CCR2. Nat Immunol 7: 311–317. 16462739 10.1038/ni1309

[44] Baluk P, Fuxe J, Hashizume H, Romano T, Lashnits E, et al (2007) Functionally specialized junctions between endothelial cells of lymphatic vessels. J Exp Med 204: 2349–2362. 17846148 10.1084/jem.20062596 PMC2118470

[45] Pflicke H, Sixt M (2009) Preformed portals facilitate dendritic cell entry into afferent lymphatic vessels. J Exp Med 206: 2925–2935. 19995949 10.1084/jem.20091739 PMC2806476

[46] Zimmerli C, Lee BP, Palmer G, Gabay C, Adams RH, et al (2009) Adaptive Immune Response in JAM-C-Deficient Mice: Normal Initiation but Reduced IgG Memory. J Immunol 182: 4728–4736. 19342649 10.4049/jimmunol.0803892

[47] Zaph C, Scott P (2003) Th1 Cell-Mediated Resistance to Cutaneous Infection with Leishmania major Is Independent of P- and E-Selectins. The Journal of Immunology 171: 4726–4732. 14568948 10.4049/jimmunol.171.9.4726

[48] Kropf P, Kadolsky UD, Rogers M, Cloke TE, Müller I (2010) The Leishmaniasis Model. Methods in Microbiology 37: 307–328.

