# Supplementary figures and images for: Blocking Junctional Adhesion Molecule C Enhances Dendritic Cell Migration and Boosts the Immune Responses against Leishmania major
Source: PLoS Pathog. 2014 Dec 4;10(12):e1004550. doi: 10.1371/journal.ppat.1004550 (PMC4256467; doi:10.1371/journal.ppat.1004550)

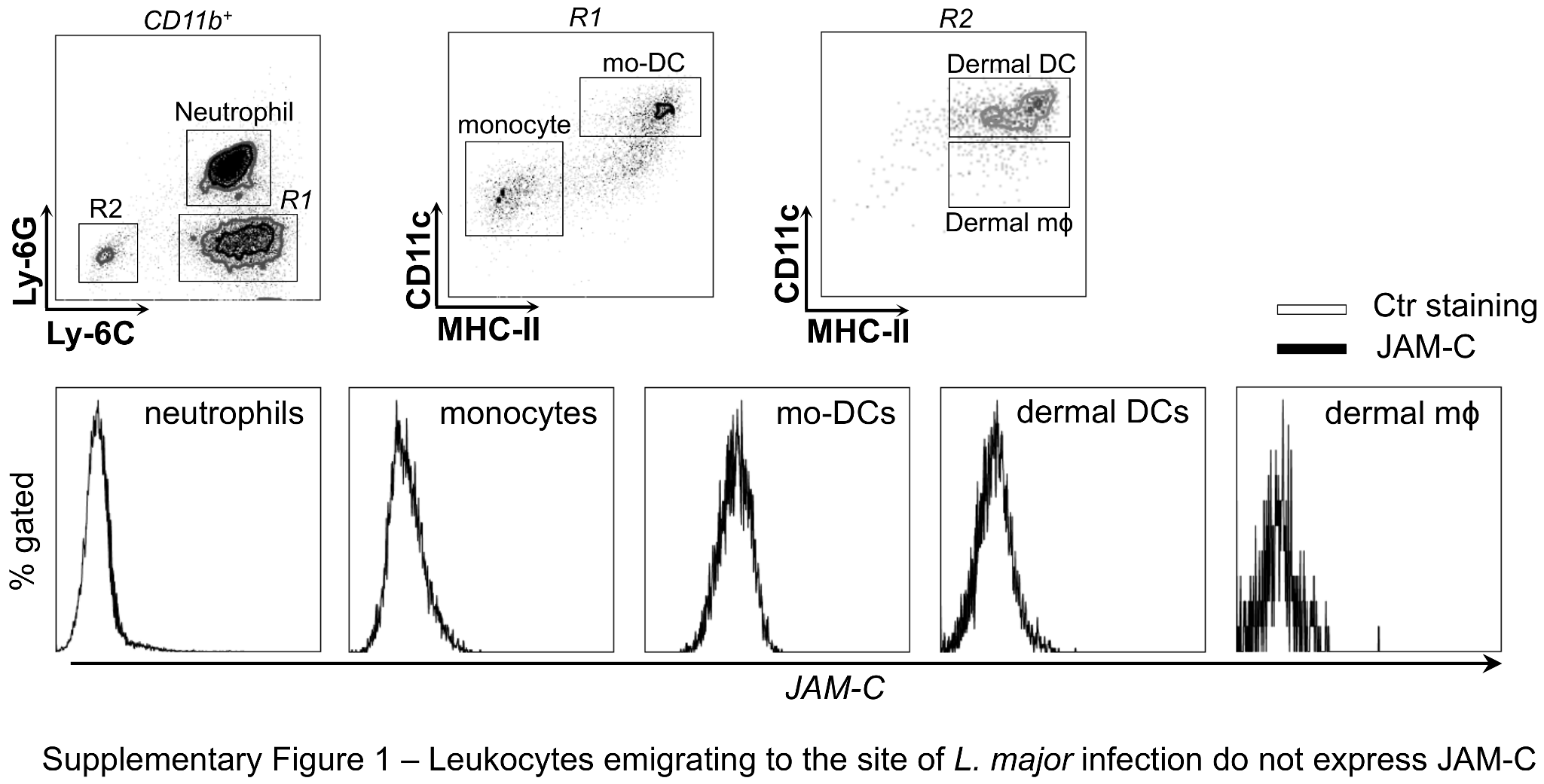

Supplement: Figure S1 — Leukocytes emigrating to the site of L. major infection do not express JAM-C. The expression of JAM-C by leukocytes emigrated from L. major infected ears was measured 24 hours post infection. CD11b+ Ly6C+ Ly6G+ represent neutrophils, CD11b+ Ly6C+ Ly6G− CD11c− IA− are monocytes, CD11b+ Ly6C+ Ly6G− CD11c+ IA+ are mo-DCs, CD11b+ Ly6C− Ly6G− CD11clow IA+ are dermal mφ, and CD11b+ Ly6C− Ly6G− CD11chigh IA+ are dermal DCs. A representative histogram overlay of JAM-C expression is shown for each population, with JAM-C staining (black line), and isotype control staining (grey line). Data are representative of two separate experiments. (TIFF) [file ppat.1004550.s001.tiff]

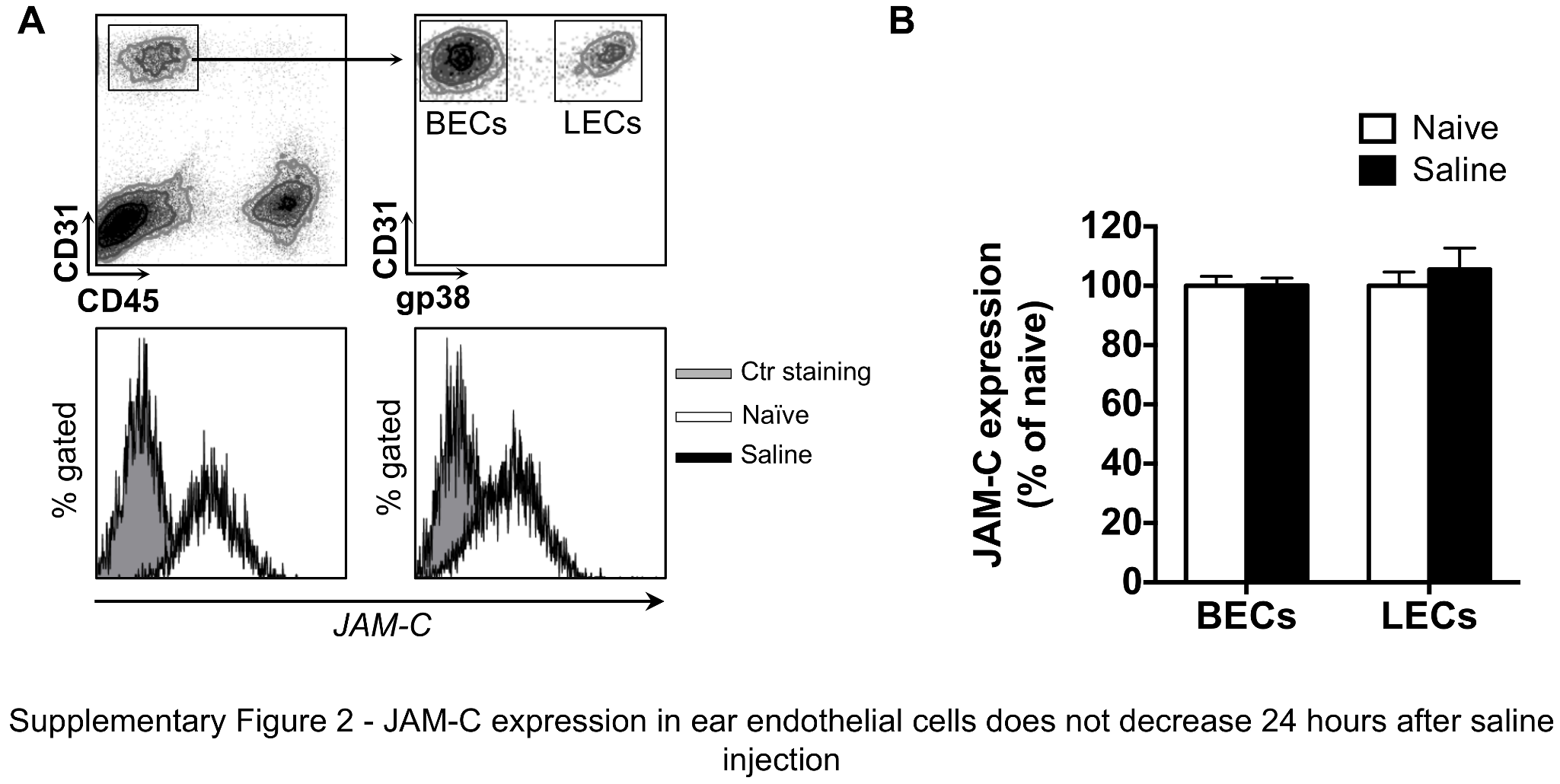

Supplement: Figure S2 — JAM-C expression in ear endothelial cells does not decrease 24 hours after saline injection (A) JAM-C levels in endothelial cells populations of mouse ear. Ears were enzymatically digested and stained for FACS analysis. CD45− CD31+ gp38− cells represent blood endothelial cells (BECs), whereas CD45− CD31+ gp38+ cells are lymphatic endothelial cells (LECs). For each population a representative histogram overlay is shown with JAM-C in endothelial cells from naïve ears (white filled), JAM-C in endothelial cells from saline injected ears (black filled), and the isotype control staining (grey filled). (B) The MFI of JAM-C in naïve mouse ears (white bars) versus saline injected mouse ears (black bars) was measured in BECs and LECs. The Y-axis scale represents MFI normalized to the mean MFI of naïve ears. Data represent the mean ± SEM of five mice per group pooled from two separate experiments. (TIFF) [file ppat.1004550.s002.tiff]

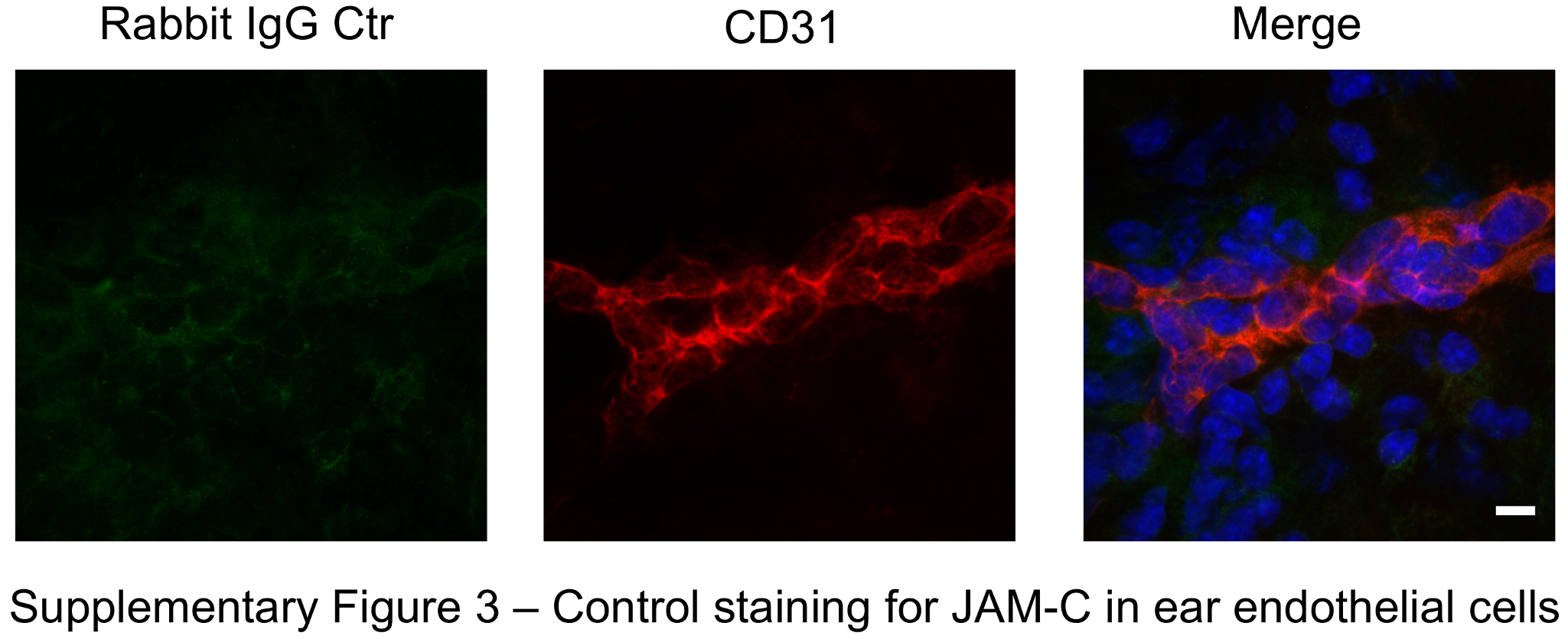

Supplement: Figure S3 — Control staining for JAM-C in ear endothelial cells. Ear sections were stained for Rabbit IgG control (green), CD31 (red). Nucleus was stained with DAPI (blue). Scale bars, 10 µm. This supporting information is related to Fig. 1D. (TIFF) [file ppat.1004550.s003.tiff]

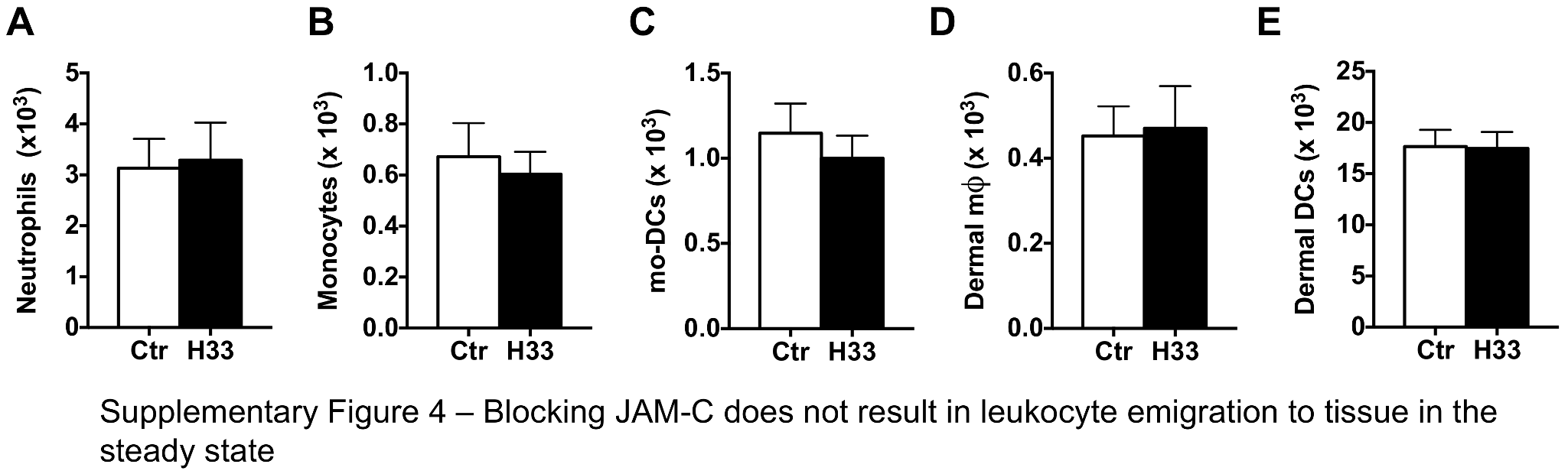

Supplement: Figure S4 — Blocking JAM-C does not result in leukocyte emigration to tissue in the steady state. The number of neutrophils (A), monocytes (B), mo-DCs (C), dermal mφ (D), and dermal DCs (E) emigrating from ears was measured in H33-treated (H33, black bar) versus isotype control-treated mice (Ctr, white bars) 24 hours after antibody administration. Data represent the mean ± SEM of fifteen mice per group pooled from 3 separate experiments, and were analyzed by the unpaired Student's t test. (TIFF) [file ppat.1004550.s004.tiff]

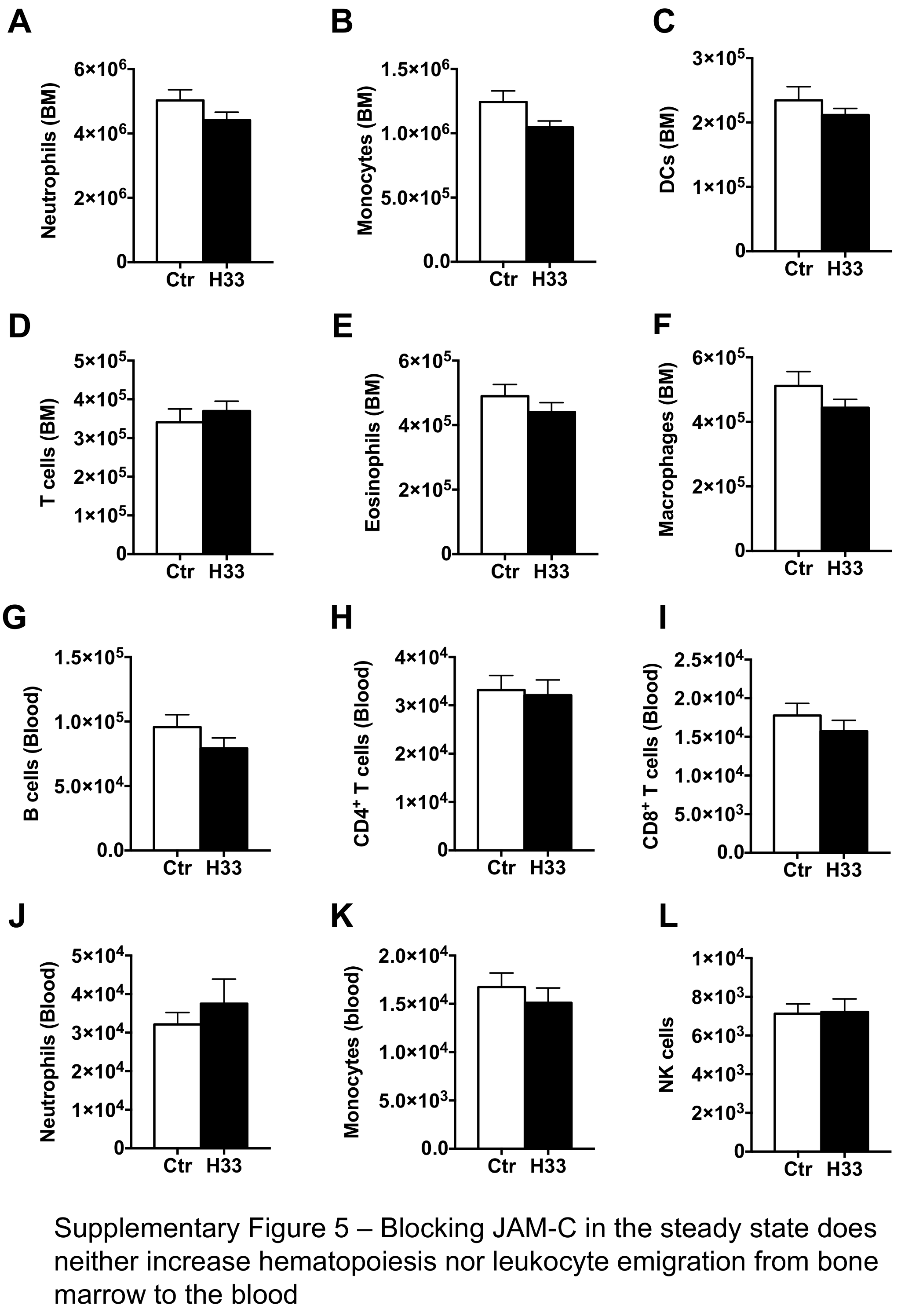

Supplement: Figure S5 — Blocking JAM-C in the steady state does neither increase hematopoiesis nor leukocyte migration from bone marrow to the blood. Naïve C57BL/6 mice were treated with H33 or isotype control antibody for 24 hours. The number of neutrophils (A), monocytes (B), DCs (C), T cells (D), eosinophils (E), and macrophages (F) from the bone marrow (BM); and B cells (G), CD4+ T cells (H), CD8+ T cells (I), neutrophils (J), monocytes (K), and NK cells (L) from blood in H33-treated (black bar) versus isotype control-treated mice (white bars) is shown. Data represent the mean ± SEM of five mice per group, and were analyzed by the unpaired Student's t test. Data are representative of three separate experiments. (TIFF) [file ppat.1004550.s005.tiff]

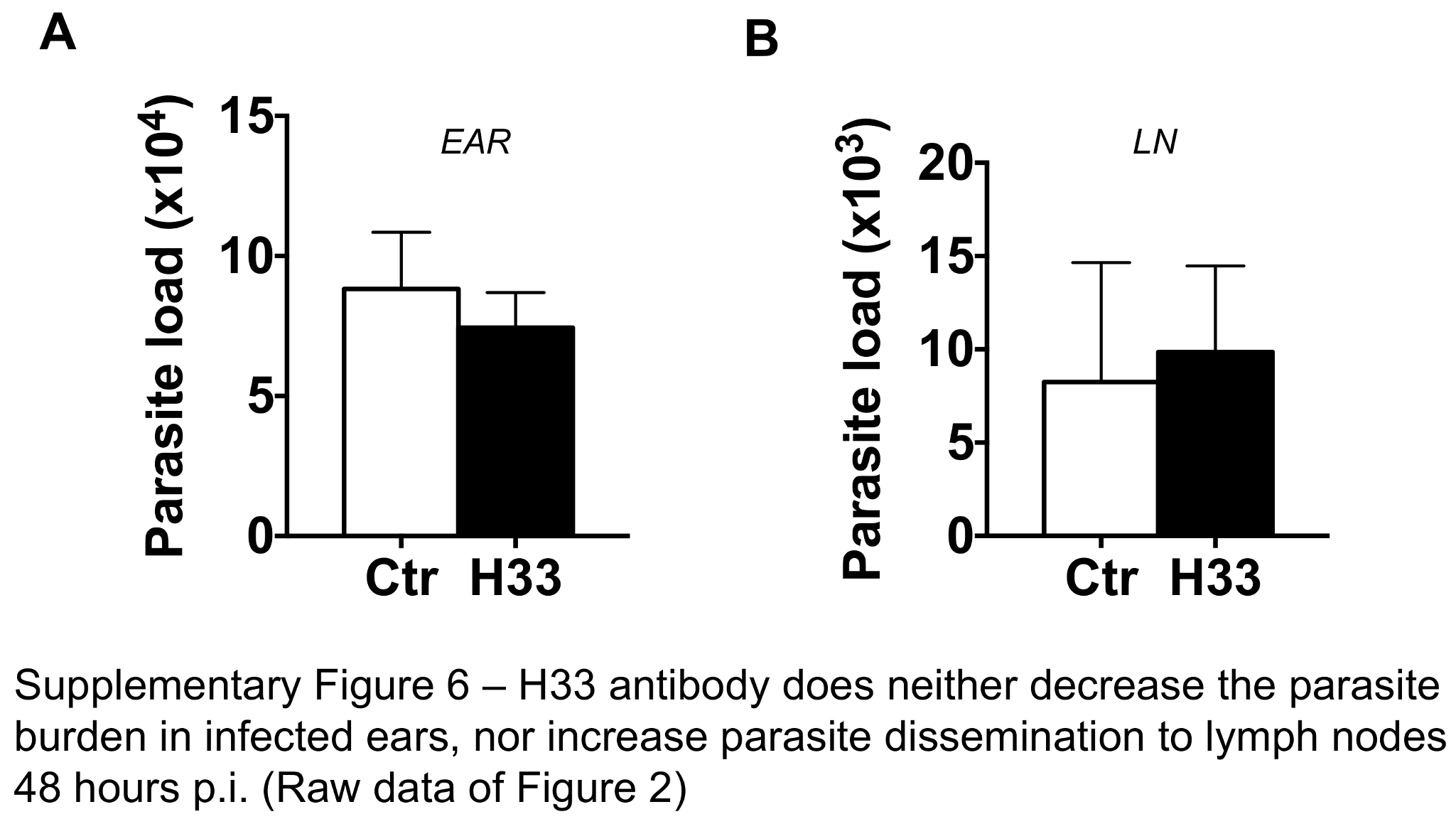

Supplement: Figure S6 — H33 antibody does neither decrease the parasite burden in infected ears, nor increase parasite dissemination to lymph nodes 48 hours p.i. (Raw data of Fig. 2 ). The parasite burden in infected ears (A) and draining lymph nodes (B) were measured 48 hours p.i. by LDA. Data represent the mean ± SEM of five mice per group from one representative experiment, and were analyzed by the unpaired Student's t test. These supporting informations are related to Fig. 2H and I. (TIFF) [file ppat.1004550.s006.tiff]

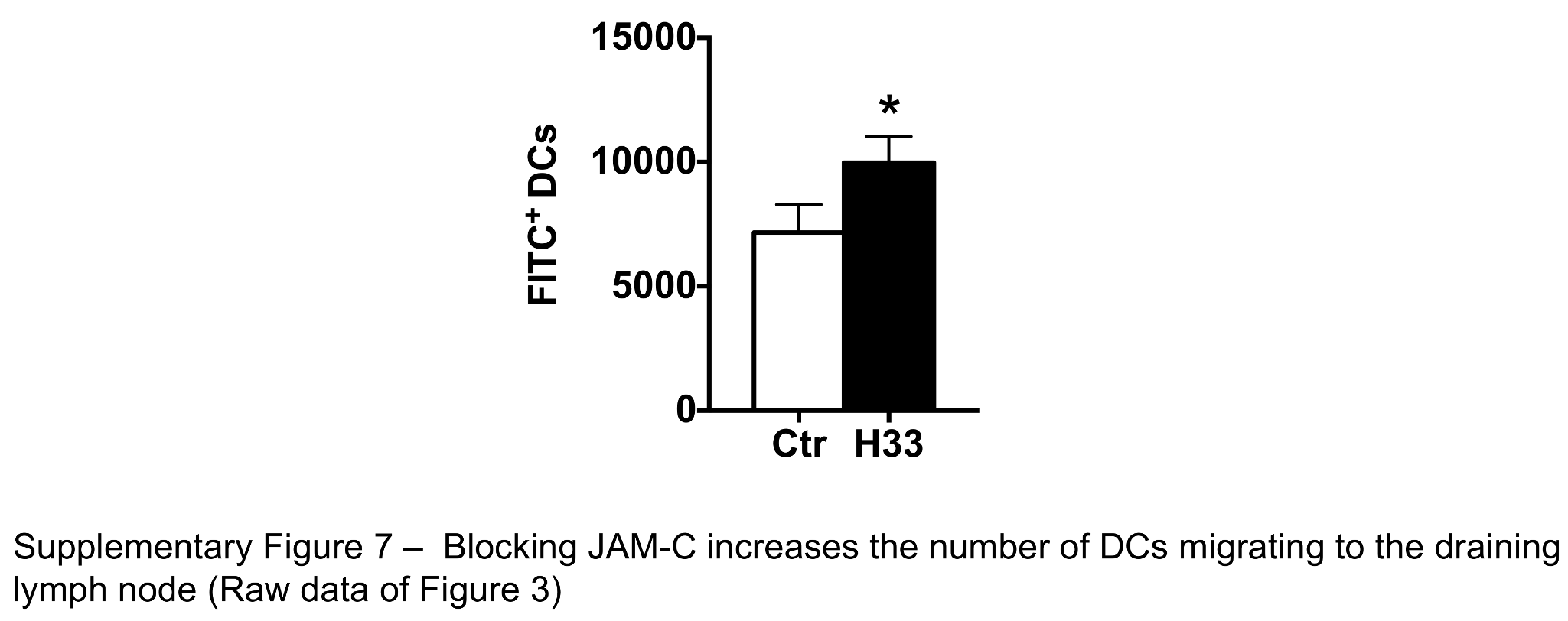

Supplement: Figure S7 — Blocking JAM-C increases the number of DCs migrating to the draining lymph node (Raw data of Fig. 3 ). The ear draining lymph nodes were harvested and stained for FACS analysis 18 hours after FITC-painting. The number of IAhigh CD11c+ FITC+ migratory DCs was counted. Data represent the mean ± SEM of six mice per group, and were analyzed by the unpaired Student's t test with *: p<0.05. This supporting information is related to Fig. 3B. (TIFF) [file ppat.1004550.s007.tiff]

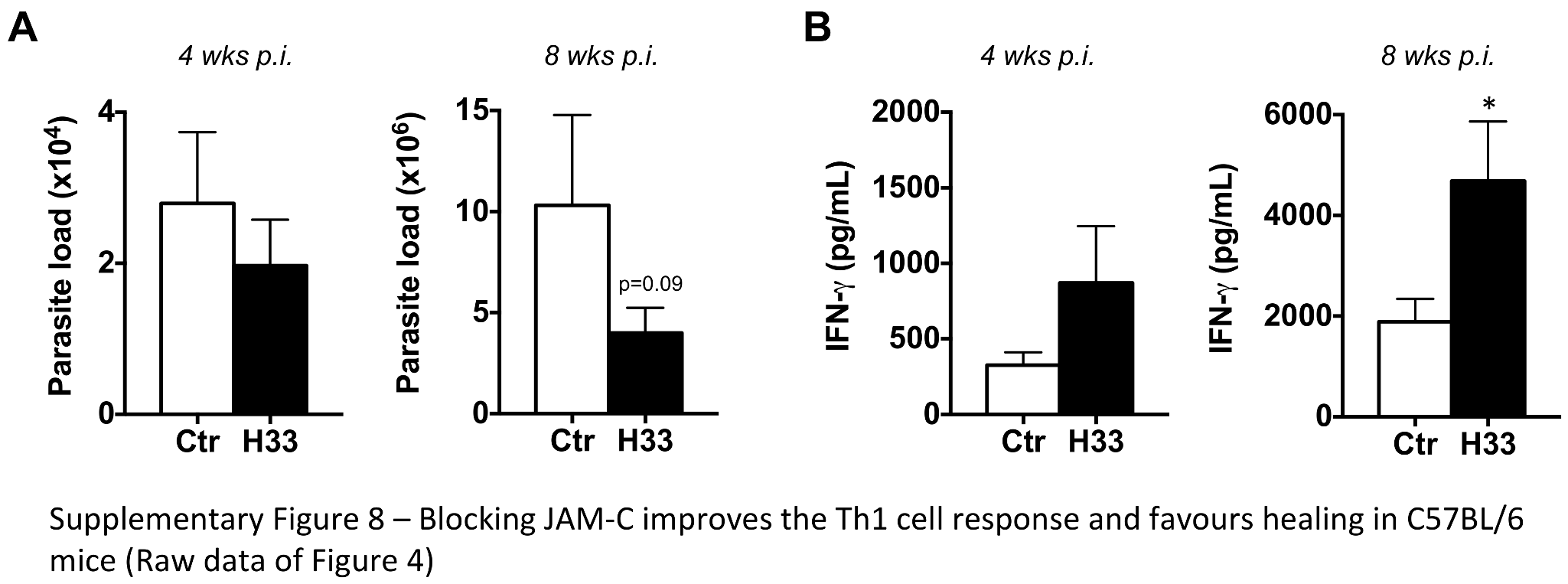

Supplement: Figure S8 — Blocking JAM-C improves the Th1 cell response and favours healing in C57BL/6 mice (Raw data of Fig. 4 ). Mice were inoculated with L. major in the ear dermis and treated with H33 or the isotype control antibody for 3 weeks, twice a week. (A) The parasite burden in infected ears was measured by LDA 4 and 8 weeks p.i. Data represent mean ± SEM of ten mice per group for both time points. (B) Draining lymph node cells were restimulated for 72 hrs with UV-irradiated L. major and the secreted IFN-γ was measured. Data represent the mean ± SEM of mice from panel A. Data were analyzed by the unpaired Student's t test with *:p<0.05. These supporting informations are related to Fig. 4B and E. (TIFF) [file ppat.1004550.s008.tiff]

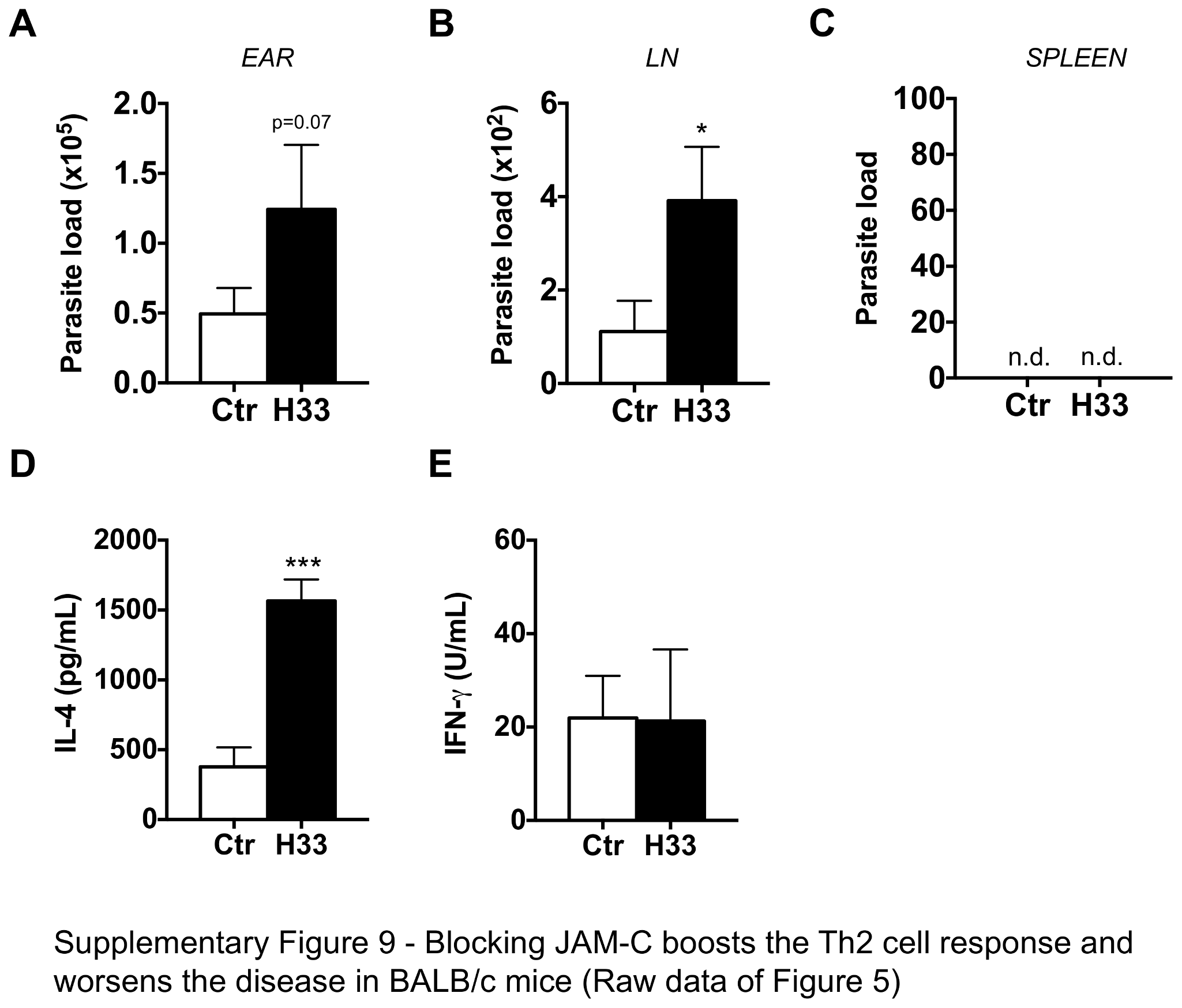

Supplement: Figure S9 — Blocking JAM-C boosts the Th2 cell response and worsens the disease in BALB/c mice (Raw data of Fig. 5 ). (A–C) Mice were inoculated with 1×10^4^ stationary phase L. major promastigotes in the ear dermis and treated with H33 or control antibody for 3 weeks. The parasite burden in infected ears (A), draining lymph nodes (B), and spleens (C) were measured by LDA. (D–E) Draining lymph nodes cells were restimulated with UV-irradiated L. major for 72 hours, and the IL-4 (D) and IFN-γ (E) produced were measured. Data represent the mean ± SEM of 5 mice per group. Data were analyzed by the unpaired Student's t test with *:p<0.05, ***: p<0.001. n.d. not-detectable. These supporting informations are related to Fig. 5G, H, K and L. (TIFF) [file ppat.1004550.s009.tiff]
